# *Leishmania*-Induced Inactivation of the Macrophage Transcription Factor AP-1 Is Mediated by the Parasite Metalloprotease GP63

**DOI:** 10.1371/journal.ppat.1001148

**Published:** 2010-10-14

**Authors:** Irazú Contreras, María Adelaida Gómez, Oliver Nguyen, Marina T. Shio, Robert W. McMaster, Martin Olivier

**Affiliations:** 1 Department of Microbiology and Immunology, McGill University, Montreal, Quebec, Canada; 2 Centre for the Study of Host Resistance and the Research Institute of McGill University Health Centre, Montreal, Quebec, Canada; 3 Department of Medical Genetics, University of British Columbia, Vancouver Hospital, Vancouver, British Columbia, Canada; National Institutes of Health, United States of America

## Abstract

*Leishmania* parasites have evolved sophisticated mechanisms to subvert macrophage immune responses by altering the host cell signal transduction machinery, including inhibition of JAK/STAT signalling and other transcription factors such as AP-1, CREB and NF-κB. AP-1 regulates pro-inflammatory cytokines, chemokines and nitric oxide production. Herein we show that upon *Leishmania* infection, AP-1 activity within host cells is abolished and correlates with lower expression of 5 of the 7 AP-1 subunits. Of interest, c-Jun, the central component of AP-1, is cleaved by *Leishmania*. Furthermore, the cleavage of c-Jun is dependent on the expression and activity of the major *Leishmania* surface protease GP63. Immunoprecipitation of c-Jun from nuclear extracts showed that GP63 interacts, and cleaves c-Jun at the perinuclear area shortly after infection. Phagocytosis inhibition by cytochalasin D did not block c-Jun down-regulation, suggesting that internalization of the parasite might not be necessary to deliver GP63 molecules inside the host cell. This observation was corroborated by the maintenance of c-Jun cleavage upon incubation with *L. mexicana* culture supernatant, suggesting that secreted, soluble GP63 could use a phagocytosis-independent mechanism to enter the host cell. In support of this, disruption of macrophage lipid raft microdomains by Methyl β-Cyclodextrin (MβCD) partially inhibits the degradation of full length c-Jun. Together our results indicate a novel role of the surface protease GP63 in the *Leishmania*-mediated subversion of host AP-1 activity.

## Author Summary

Leishmaniasis is a tropical disease affecting more than 12 million people around the world. The disease is caused by the *Leishmania* parasites that are transmitted to the mammalian host by a sandfly vector when it takes a blood meal. The parasites are able to survive and multiply inside of cells that comprise the primary defence of the host, the macrophages. We have extensively studied the mechanism whereby *Leishmania* escapes from macrophage microbicidal functions. Herein we report that the parasite can inactivate these cells by decreasing the activity of transcription factors such as Activated Protein-1(AP-1) that are involved in transcription of genes coding for antimicrobial functions of macrophages. In this study, we showed that *Leishmania* parasites use their most abundant surface protein GP63 to inactivate the AP-1 transcription factor. Furthermore, we found that GP63 enter into the macrophages independently of parasite internalization using lipid rich microdomains localized in the cellular membrane. In addition, GP63 was observed to reach the nuclear compartment where it cleaves AP-1 subunit proteins. Collectively, our findings reveal a novel mechanism used by *Leishmania* to facilitate its survival and propagation within its mammalian host cells. Better knowledge concerning the mechanisms whereby this pathogen can escape the innate immune response may help to develop new anti-*Leishmania* therapy.

## Introduction

Parasites of the *Leishmania* genus are the causative agent of leishmaniasis; a disease distributed worldwide affecting more than 12 million people in 88 countries [1]. Leishmaniasis is a complex of diseases ranging from self-healing cutaneous lesions to lethal visceral afflictions [2]. In its mammalian host, *Leishmania* is an obligate intracellular pathogen infecting hematopoietic cells of the monocyte/macrophage lineage. Macrophages are specialized for the destruction of invading pathogens and priming the immune response. In order to survive within these cells, *Leishmania* has evolved sophisticated mechanisms to subvert macrophage microbicidal functions such as inhibition of nitric oxide (NO) production and cytokine-inducible macrophage functions [3]. This occurs as the direct consequence of parasite-mediated activation of protein tyrosine phosphatases, alteration of signal transduction and inhibition of nuclear translocation and activity of transcription factors such as NF-κB, STAT, CREB and AP-1[4], [5]. Activated Protein-1 (AP-1) is an important transcription factor that mediates gene regulation in response to physiological and pathological stimuli, including cytokines, growth factors, stress signals, bacterial and viral infections, apoptosis, as well oncogenic responses [6], [7]. AP-1 is formed by homodimers of Jun family members (c-Jun, Jun B and Jun D), or heterodimers of Jun and Fos family members (c-Fos, Fos B, Fra 1 and Fra 2). Homodimers within the Fos family do not occur due to conformational repulsion [8].

Previous studies have reported that the AP-1 transcription factor is inactivated by *Leishmania* infection. For instance, activation of macrophage AP-1 and NF-κB is inhibited by *L. donovani* promastigotes through an increase in intracellular ceramide concentration, which leads to the down-regulation of classical PKC activity, up-regulation of calcium independent atypical PKC-ζ and dephosphorylation of Extracellular Signal-Regulated Kinases (ERK) [9], [10]. Other studies have shown that *Leishmania* alters signal transduction upstream of c-Fos and c-Jun by inhibiting ERK, JNK and p38 MAP Kinases, resulting in a reduction of AP-1 nuclear translocation [11], [12]. However, little is known about the molecular mechanism (s) by which *Leishmania* parasites are able to inactivate this important transcription factor.

Many *Leishmania*-specific factors such as lipophosphoglycan (LPG), A2 proteins, cysteine peptidases (CPs) and the protease GP63, contribute to *Leishmania* virulence and pathogenicity. LPG has been implicated in altering phagosome maturation in *L. donovani* infection [13].The A2 proteins of *L. donovani* are involved in intracellular amastigote survival [14].The cysteine peptidases of *L. mexicana* are implicated in facilitating the survival and growth of the parasite [15]. Furthermore GP63, also known as the major surface protease (MSP), has been related to resistance to complement-mediated lysis, among others [16], [17]. GP63 is a metalloprotease which belongs to the metzincin class. It is the most abundant surface glycoprotein of the parasite and accounts for 1% of the total protein content of *L. mexicana* promastigotes [18]. GP63 of different *Leishmania* species encode similar amino acid sequences, although slight substrate specificity variations have been reported [19]. Specific characteristics of this class of metalloproteases include a conserved signature motif HEXXHXXGXXH and an N-terminal pro-peptide that serves to maintain the pro-enzyme inactive during translation, which is removed upon protein maturation and activation [20]. The mature GP63 contains 3 domains: 1) N-terminal (bases ∼101-273) which comprises a structure corresponding to the catalytic module of metzincin class zinc protease, 2) central domain (bases ∼274–391) and 3) C-terminal domain containing the site of glycosylphosphatidylinositol (GPI) anchor addition (bases ∼392–577) [17], [18], [20], [21]. We have previously shown that this protease actively participates in the cleavage of NF-κB [5], protein tyrosine phosphatases (PTP) [22] and actin cytoskeleton regulators [23]. In this study we have investigated how GP63 contributes to the inactivation of AP-1 and the degradation of its subunits. Herein, we report that GP63 enters the host cell via lipid raft microdomains, independently of parasite internalization, and for the first time show that it is able to reach the nuclear compartment shortly after infection where it degrades and cleaves c-Jun and other AP-1 subunits.

## Results

### Alteration of steady state level of AP-1 upon *Leishmania* infection involves down-regulation/degradation of selected Jun/Fos family members

We have previously studied the effect of *Leishmania* promastigote infection on the activity of various macrophage transcription factors: STAT-1α degradation is proteasome and receptor-dependent and is mediated through a mechanism involving PKC-α [4], and cleavage of NF-κB subunits upon *Leishmania* infection is in part dependent on GP63 [5]. As AP-1 is an important transcription factor regulating the expression of many genes involved in the activation of macrophage functions (*TNFα*, *iNOS*, and *IL-12*) [24], [25], [26] critical for the adequate innate immune response against *Leishmania* infection, we investigated the mechanisms underlying AP-1 inactivation upon *Leishmania* infection.

**Figure 1:**
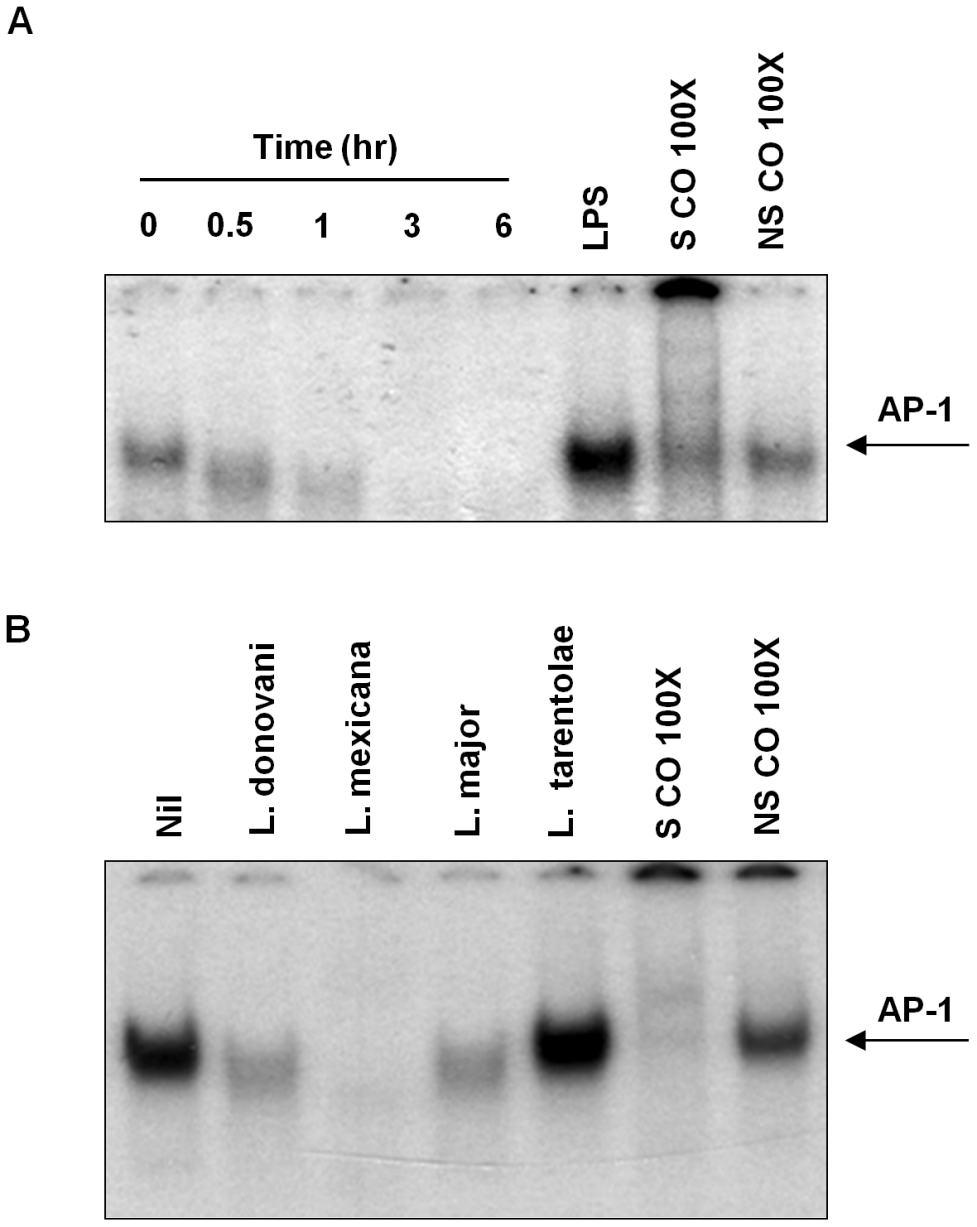
Infection with different species of *Leishmania* inhibits AP-1 DNA binding activity. (**A**) B10R macrophages were infected for 0.5, 1, 3 and 6 hr with *L. donovani*, at a ratio 20∶1 parasite/macrophage. Nuclear proteins were isolated and EMSA for AP-1 DNA binding activity was performed. A consensus DNA sequence for SP-1 binding was used as non-specific competitor (NSCO). A 100× molar excess of AP-1 probe was used as a specific competitor (SCO). (**B**) B10R macrophages were infected for 1 hr with L. *mexicana*, *L. major*, *L donovani infantum* or *L. tarentolae* and treated as in (**A**).

To evaluate nuclear translocation and DNA binding activity of macrophage AP-1 upon infection with *Leishmania* promastigotes, Electrophoretic Mobility Shift Assays (EMSA) were performed. As shown in Figure 1A, AP-1 nuclear translocation was inhibited as early as 30 min post-infection in *L. donovani*-infected macrophages. Furthermore, we observed that other mammalian pathogenic *Leishmania* species (*L. mexicana* and *L. major*) were able to alter AP-1 DNA binding (Figure 1B). Of interest, we did not observe any effect on macrophages infected with *L. tarentolae*, whose pathogenicity is limited to reptilian hosts.

**Figure 2:**
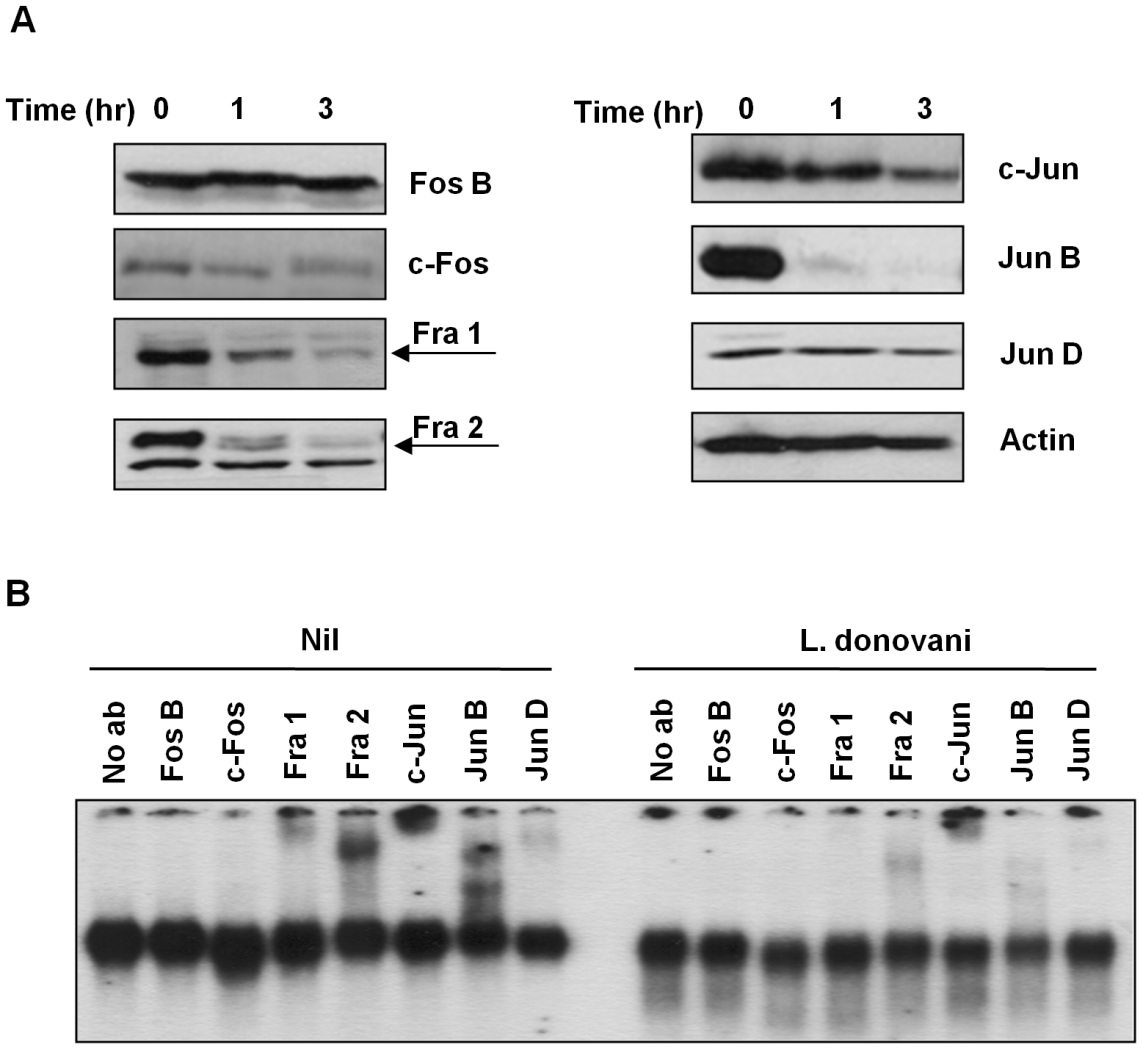
AP-1 subunits are degraded after infection with *Leishmania* parasites. (**A**) Western blot analysis of AP-1 subunit proteins extracted from B10R macrophages infected with *L. donovani* for 1 and 3 hr. β-actin was used as a loading control. (**B**) Super shift assays of B10R macrophages infected with *L. donovani* for 1 hr. Nuclear proteins were super shifted using antibodies against c-Jun, Jun B, Jun D, c-Fos, Fos B, Fra 1 and Fra 2 AP-1 subunits.

**Figure 3:**
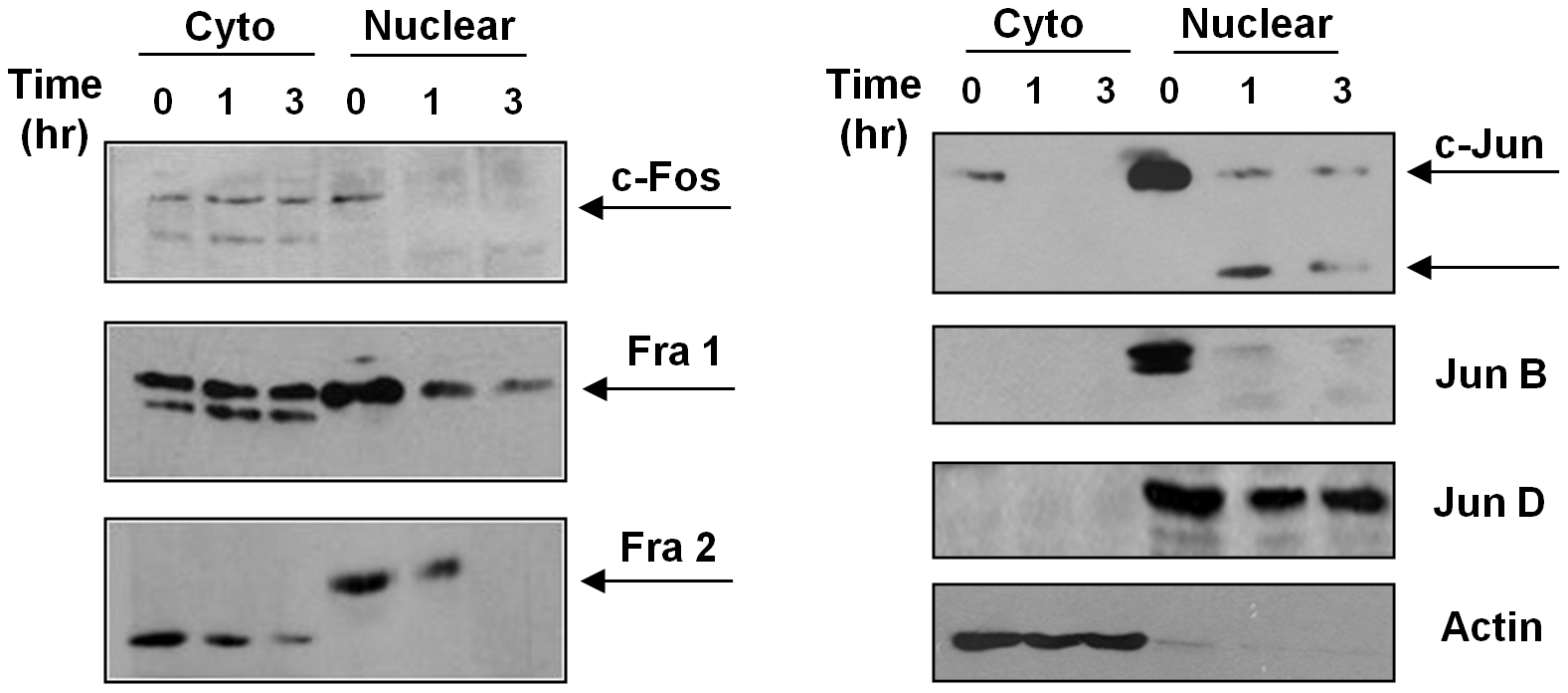
Subcellular localization of AP-1 subunits. B10R macrophages were infected with *L. donovani* for 1 and 3 hr. Cytoplasmic and nuclear distribution of the AP-1 subunits was monitored by Western Blot analysis. β-actin was used as a loading control for cytoplasmic fraction, and Jun D for nuclear fraction.

In order to better understand the observed decrease in AP-1 activity, we performed Western Blot (WB) analysis in the total cell extracts to evaluate the various AP-1 subunits during infection. Five out of the seven AP-1 subunits (c-Fos, Fra 1, Fra 2, c-Jun and Jun B) showed decreased expression after infection with *L. donovani*, whereas Jun D presented a slight reduction and Fos B was maintained intact (Figure 2A). To further confirm the presence of these subunits in the AP-1 complex we used super shift analysis. This approach uses the incorporation of specific antibodies to nuclear protein extracts, allowing the visualization of the antibody: protein: DNA complexes by retarding the migration of the specific bands in the gel. As shown in Figure 2B, inclusion of antibodies specific for Fos B, c-Fos, Fra 1, Fra 2, c-Jun, Jun B and Jun D, demonstrated presence of Fra 1, Fra 2, c-Jun, Jun B and Jun D, but not c-Fos or Fos B, within the macrophage nuclear AP-1 complex. Importantly, *L. donovani* infection clearly affected the AP-1 complex as the bands observed for Fra-1, Fra-2, c-Jun, Jun B and Jun D in the super shift assay was greatly reduced. Whereas the c-Fos protein was not detectable by super shift assay, this protein was still affected by *Leishmania* infection since less expression was observed by WB (see Figures 2A and 3), suggesting that the amount of c-Fos might not be enough to be detected by super shift assay.

After phosphorylation AP-1 subunits are translocated into the nucleus where they dimerize with another subunit to form an active AP-1 complex [6], [7], [8], [27]. To determine the level of expression of each AP-1 subunit in the different cellular compartments (cytoplasm *vs* nucleus), we performed WB analysis on separated nuclear and cytoplasmic fractions. As shown in Figure 3, different phenomena can be observed. c-Fos and Fra-1 expression in the cytoplasmic fraction are not altered with *Leishmania* infection, but their expression in the nuclear fraction is decreased in infected macrophages; Fra-2 and c-Jun have decreased expression in both cytoplasmic and nuclear fractions, and Fra-2 in the nuclear fraction presents a band with less migration than the band observed in the cytoplasmic fractions, possible due to post-nuclear translocation modifications. On the other hand, Jun-B and Jun-D were detected only in the nuclear fraction; however, only Jun-B expression is affected by *Leishmania* infection. The lower expression of the different subunits in the nucleus could be due to decreased complex formation and/or cleavage and further degradation of the subunits, as it is possible to detect smaller bands (c-Jun and Jun-B).

### *Leishmania* major surface protease GP63 is involved in AP-1 inactivation

**Figure 4:**
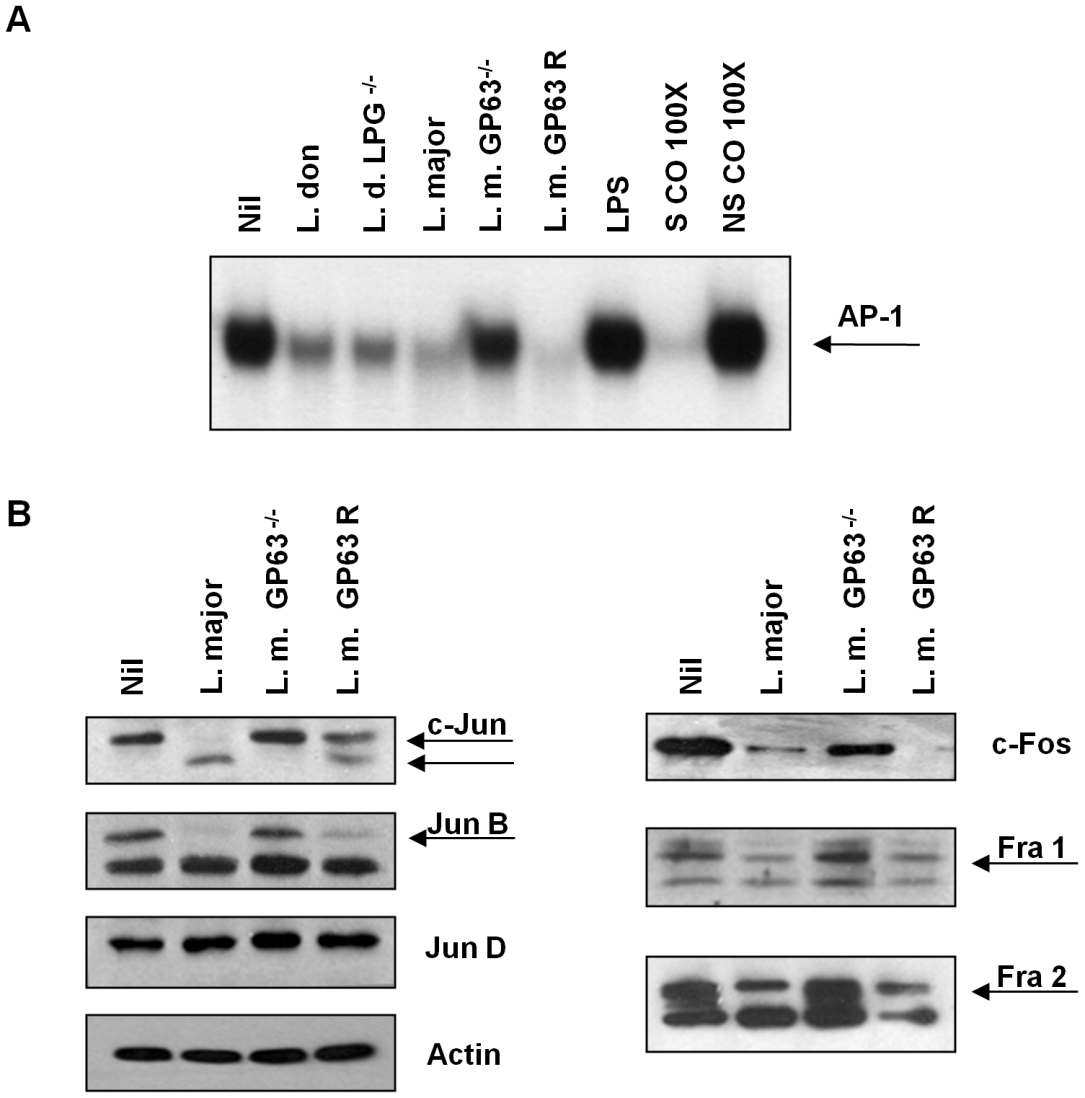
Role of *Leishmania* surface molecules in the inactivation of AP-1. (**A**) EMSA for AP-1 DNA binding activity of nuclear extracts from B10R macrophages infected for 1 hr with *L. donovani* 1S2D (LPG^+/+^), *L. donovani* LPG^−/−^, *L. major* (WT), *L. major* GP63 ^−/−^ or *L. major* GP63 Rescued (GP63 R) promastigotes. A consensus DNA sequence for SP-1 binding was used as non-specific control (NSCO). A 100× molar excess of AP-1 probe was used as a specific competitor (SCO). 1 hr stimulation with LPS (100 ng/ml) was used as a positive control for the induction of AP-1 DNA binding. (**B**) Macrophages were infected for 1 hr with *L. major* (WT), *L. major* GP63 ^−/−^ or *L. major* Rescued promastigotes at 20∶1 ratio. WB of AP-1 subunits was performed with the total cell lysate. β-actin was used as a loading control.

*Leishmania* surface molecules such as LPG and GP63, among others, play important roles as virulence factors and modulators of host cell signalling. LPG, for instance, has been implicated in the interference of phagolysosome maturation and inactivation of PKC signalling [13], [28]. GP63 has been related to resistance to complement-mediated lysis, migration of *Leishmania* parasites through the extracellular matrix by degradation of casein, fibrinogen and collagen [16], [21] and inhibition of JAK/STAT signalling by modulation of PTP activities [22]. To address the role of LPG and GP63 in AP-1 inactivation we performed EMSA with extracts from cells infected with *Leishmania* mutants for these two surface molecules. As shown in Figure 4A, LPG is not involved in the AP-1 degradation induced by *Leishmania* infection since DNA binding in macrophages infected with either *L. donovani* or *L. donovani* LPG^−/−^ promastigotes was similarly altered. Importantly, however, we observed that cells infected with an *L. major* strain lacking GP63 (*L. major* GP63^−/−^) [29] showed normal AP-1 DNA binding capacity, compared to uninfected controls. This suggests that GP63 but not LPG is highly involved in the mechanism responsible for the inactivation of AP-1 transcription factor.

To further elucidate the role of GP63, we performed WB analysis of all the AP-1 subunits of macrophages infected with *L. major*, *L. major* GP63^−/−^ and *L. major* GP63 Rescued. Results obtained further revealed that in the absence of GP63, no degradation or cleavage of any AP-1 subunit was evident (Figure 4B); supporting the finding that AP-1 activity is unaffected in *L. major* GP63^−/−^-infected macrophages.

In addition, to validate the role of GP63 on AP-1 activity we verified the expression of IL-12 transcripts, as it is known that AP-1 regulates its transcription [24]. As shown in the Figure S1, LPS-induced IL-12 expression is fully blocked by all infectious *Leishmania* species but not by *L. major* GP63^−/−^ and *L. tarentolae*. As expected, the JNK/c-Jun inhibitor has completely inhibited LPS-induced IL-12 transcripts.

### GP63 action requires macrophage lipid raft and is not dependent on parasite phagocytosis

**Figure 5:**
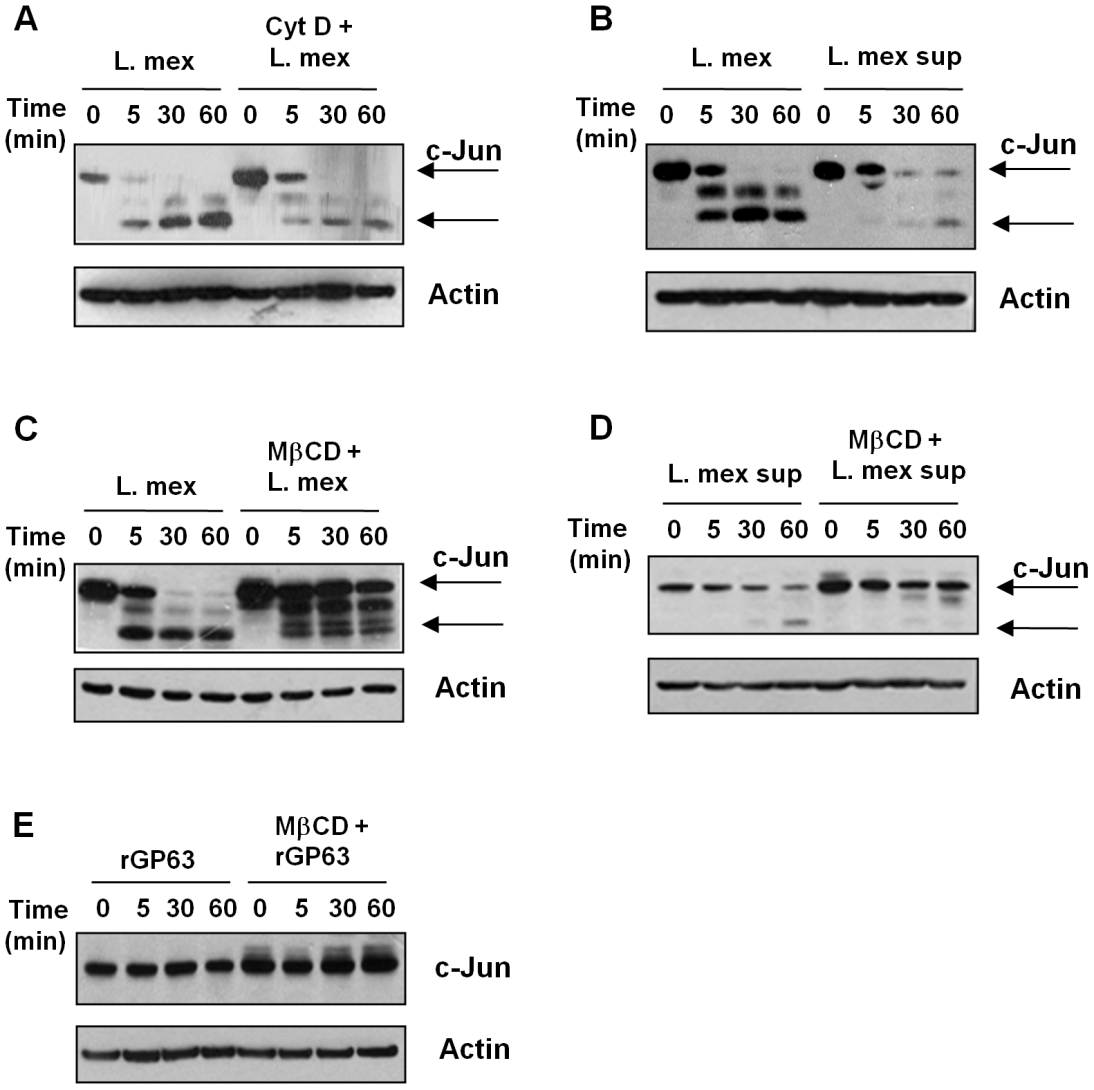
GP63 delivery into the host cell is mediated via lipid raft. (**A**) B10R macrophages were pre-treated or not with 2 µM cytochalasin D for 1 hr and then infected with *L. mexicana* for indicated times. (**B**) B10R macrophages were incubated with either with whole parasite or culture supernatant of *L. mexicana* promastigotes. Macrophages were pre-treated or not with 20 mM of methyl β-ciclodextrin (MβCD) for 1 hr and infected with *L. mexicana* promastigotes (**C**), incubated with *L. mexicana* supernatant (**D**) or recombinant GP63 (rGP63) (**E**). For all the Figures, total cell extracts and β-actin as a loading control were used.

*Leishmania* GP63 can be found in three different forms: 1) Intracellular GP63, 2) Surface GPI-anchored GP63 and 3) secreted or released GP63 [21], [30]. For GP63 to target its intracellular macrophage substrates, it needs to gain access to or be internalized by the macrophage. To explore whether the internalization of the parasite is necessary to deliver GP63 inside the cell, murine macrophages were pre-treated with the phagocytosis inhibitor cytochalasin D which inhibits actin polymerization, therefore blocking internalization by phagocytosis (Figure S2A). We used c-Jun as a model protein to evaluate the cleavage and degradation of the AP-1 subunits. WB analysis showed that parasite phagocytosis was not necessary for c-Jun cleavage and less expression (Figure 5A). To confirm this, we incubated macrophages with the culture supernatant of *L. mexicana* promastigotes, which is rich in soluble GP63 [18], [31]. WB showed that even in the absence of the parasite, c-Jun degradation was observed (Figure 5B).

Since phagocytosis seems not to be completely required in the internalization of GP63 we addressed whether GP63 internalization could be dependent on lipid raft-mediated endocytosis, given the fact that GP63 is an excreted and membrane-GPI anchored protein. On the other hand, lipid raft microdomains are highly dynamic membrane domains rich in cholesterol and sphingolipids, and present high affinity for proteins containing GPI anchors [32], [33], [34]. In order to examine the possible role of host lipid raft microdomains in GP63 internalization, we pre-treated cells with a non-cytotoxic dose (Figure S2B) of the cholesterol chelator and inhibitor of lipid raft integrity methyl-β-clyclodextrin (MβCD) prior to infection. As shown in Figure 5C, full length c-Jun was not degraded in cells infected under these conditions, although interestingly, a cleavage fragment was still observed. Pre-treatment of macrophages with MβCD and subsequent incubation with *L. mexicana* supernatant showed that lipid raft disruption altered internalization of parasite-free soluble GP63 and also impaired c-Jun degradation (Figure 5D). As shown in Figure S3, confocal microscopy confirmed an interaction between lipid raft microdomains and GP63, since in macrophages infected with *L. major*, GP63 (green) partially co-localized with the lipid raft marker Choleratoxin B (red). Furthermore, we have previously shown that, pre-treatment with MβCD before infection abrogates GP63 internalization [22]. To determine if MβCD had any effect over the c-Jun expression we performed a time course analysis of macrophages stimulated with MβCD. As shown in Figure S4A, there was no alteration in the expression of c-Jun after 2 hr of incubation of the macrophages with the drug. Together these data strengthen the hypothesis that GP63 uses lipid raft microdomains for internalization independent of parasite entry.

To evaluate the role of the GPI anchor in mediating GP63 internalization via lipid raft microdomains, macrophages were incubated with a GPI-deficient recombinant GP63 (rGP63) and c-Jun degradation was monitored. WB analysis evidenced that neither degradation nor cleavage of c-Jun occurred (Figure 5E) in the presence or absence of MβCD, similarly to what we have previously shown for GP63-mediated PTP cleavage. Moreover, although rGP63 is still internalized in macrophages to a limited extent, perinuclear localization was never detected [22].

To demonstrate that the less expression and cleavage of c-Jun observed in this set of experiments were occurring inside the cells and not as an effect of proteolysis during the preparation of the lysates, we included two experiments as controls; first, we lysed the cells using sample loading buffer 1× and the samples were boiled right after, to stop the proteolysis; second, we added 1 mM of phenanthroline (a Zn chelator [35]) to the lysis buffer to abrogate post-infection GP63 activity. In both experiments, we observed that cleavage of c-Jun under these conditions still occurs, suggesting that the cleavage of c-Jun occurs inside the cell and not during the sample preparation (Figure S4B and S4C). In addition, to establish whether GP63 proteolytic activity is critical for c-Jun cleavage in the macrophage, *L. mexicana* culture supernatant was treated with the GP63 inhibitor phenanthroline prior to its incubation with macrophages. As shown in the Figure S4D, phenanthroline fully inhibited GP63-mediated c-Jun degradation.

### GP63-mediated c-Jun cleavage occurs at perinuclear compartment

**Figure 6:**
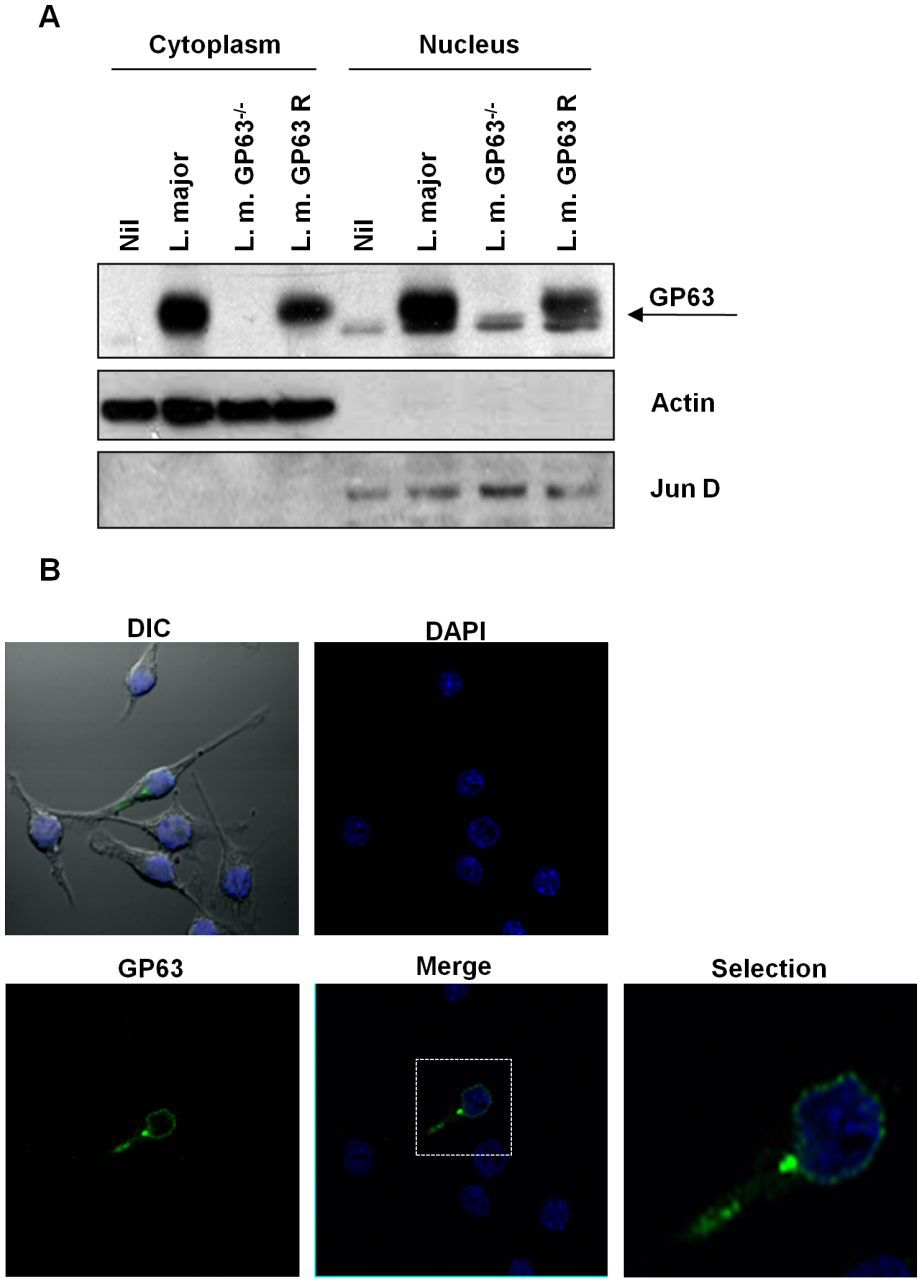
Subcellular localization of GP63. (**A**) B10R macrophages were infected for 1 hr with *L. major* (WT), *L. major* GP63^−/−^ or *L. major* GP63 Rescued and GP63 distribution in the cytoplasmic and nuclear extracts was monitored by WB. β-actin and Jun B were used as fractioning controls. (**B**) B10R macrophages were infected for 1 hr with *L. major* (WT). GP63 is shown in green and nuclei were stained with DAPI (blue).

One of the most surprising elements of the evidence presented above was the fact that GP63 is able to act on its substrate proteins within the nucleus of its host cell. In order to further demonstrate that GP63 reaches the nucleus, we separated cytoplasmic and nuclear proteins from macrophages infected with *L. major*, *L. major* GP63^−/−^ and *L. major* GP63 Rescued. WB analysis using an anti-GP63 antibody revealed that this protease is present in both fractions of *Leishmania*-infected cell extracts. As expected, there was no GP63 in macrophages infected with *L. major* GP63 ^−/−^ or the uninfected control (Figure 6A). Confocal microscopy of *Leishmania-*infected macrophages confirmed that GP63 reaches the nuclear membrane as early as 1 hr post-infection (Figure 6B). In order to demonstrate the purity of our fractions, we performed WB of the cytoplasmic and nuclear proteins against the lysosomal marker LAMP-1, the ER specific marker (the KDEL protein - Lys-Asp-Glu-Leu endoplasmic reticulum protein retention receptor), histone 2B (nuclear marker), and actin (cytoplasm marker). Figure S5 shows that actin, LAMP-1 and KDEL are only present in the cytoplasmic fraction, in contrast, histone is only detected in the nuclear fraction, and this way we are confident to say that GP63 was present in both protein fractions.

**Figure 7:**
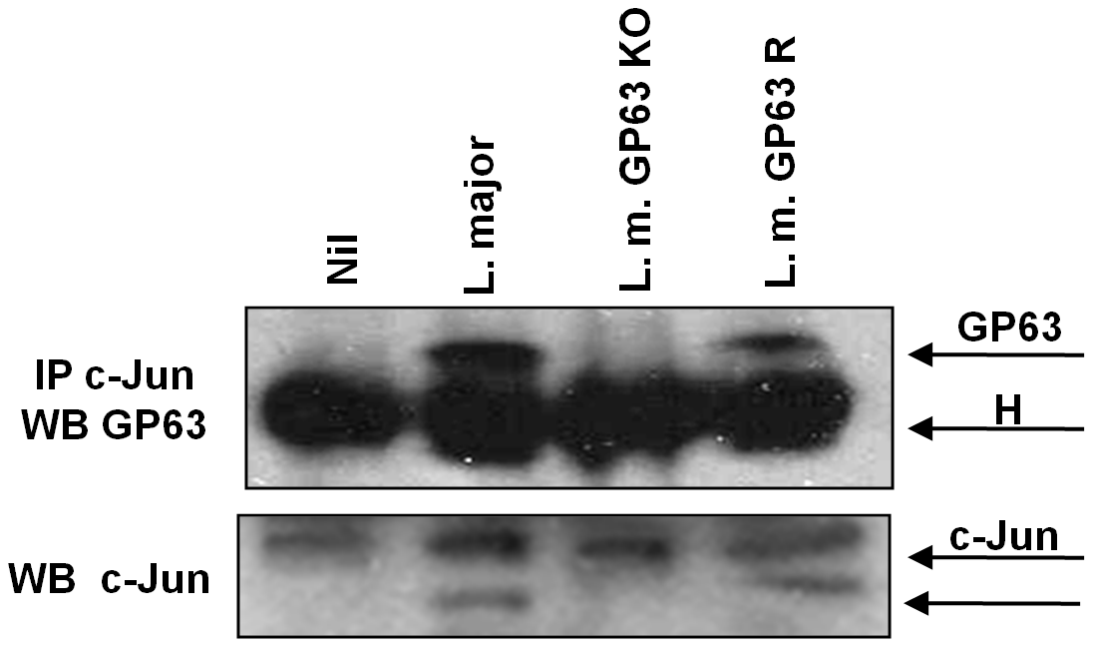
c-Jun interacts with GP63 in nuclear fraction. Proteins from nuclear extracts of macrophages infected for 1 hr with *L. major* (WT), *L. major* GP63 ^−/−^ and *L. major* GP63 Rescued (GP63 R) and nuclear proteins were immunoprecipitated using an anti-c-Jun antibody. GP63 and c-Jun co-immunoprecipitation was evaluated by western blot. H denotes the heavy chain of the immunoglobulin.

In order to confirm nuclear interaction of GP63 with c-Jun we performed a Co-Immunoprecipitation (IP) assay. c-Jun was immunoprecipitated from nuclear extracts of *Leishmania*-infected macrophages and subjected to WB analysis of GP63. This result revealed a band around 65 kDa, confirming the interaction between nuclear c-Jun and GP63 in the macrophages infected with *L. major* and *L. major* GP63 Rescue, but not with *L. major* GP63 ^−/−^ as is shown in Figure 7.

**Figure 8:**
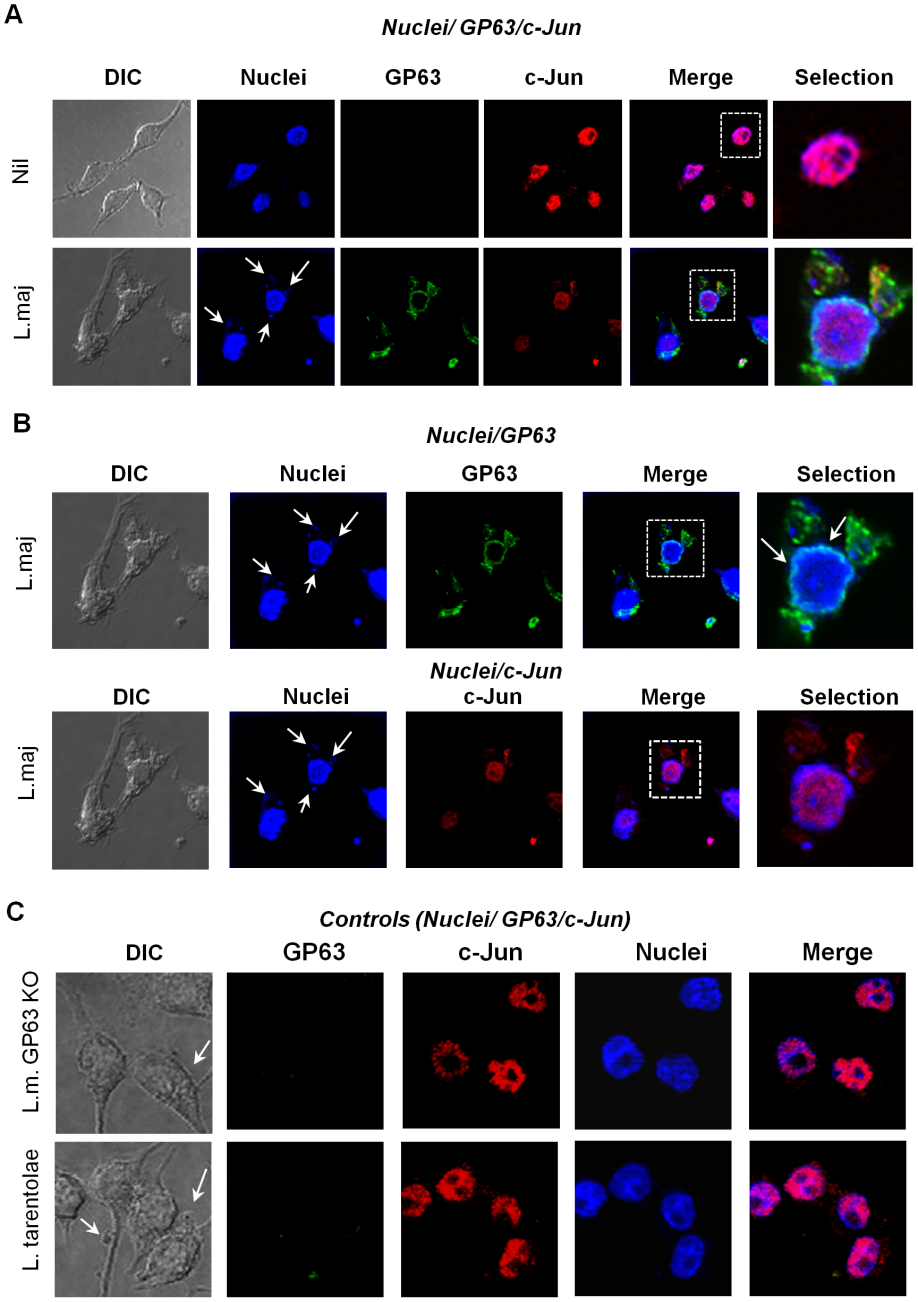
GP63 is localized in the perinuclear compartment. (**A**) Confocal microscopy images of B10R macrophages showing co-localization of nuclei (blue), c-Jun (red) and GP63 (green) in non-infected (upper panel) and cells infected for 1 hr with *L. major* (lower panel). (**B**) Confocal microscopy analysis to evaluate nuclear localization of GP63 (green) and nuclear distribution (upper panel) and degradation of c-Jun (red) in macrophages infected 1 hr with *L. major* (lower panel). Blue shows cell nuclei. (**C**) co-localization of nuclei (blue), c-Jun (red) and GP63 (green) in B10R macrophages infected for 1 hr with *L. major* GP63^−/−^ or *L. tarentolae*.

To further support that degradation of c-Jun could occurs in the nucleus we performed confocal microscopy. As shown in Figure 8A (upper panel), c-Jun (red) is localized inside the nucleus in uninfected cells. However, after 1 hr of infection GP63 was detected in the perinuclear area and the fluorescence intensity of c-Jun was considerably diminished (Figure 8A, lower panel), such reduction in the fluorescence was not observed in macrophages infected either with *L. major* GP63^−/−^ or *L. tarentolae* (Figure 8C upper and lower panels, respectively). The upper panel of Figure 8B shows partial co-localization between the nuclear stain (blue) and GP63 (green) in the periphery of the nucleus, giving a light blue signal. Of utmost importance, the panel representing c-Jun (red) versus nucleus (blue) co-localization, clearly reveals that c-Jun is absent from perinuclear area as this one is solely stained in blue (Figure 8B, lower panel). To discard possible unspecific signals in the confocal micrographs we included specific isotype and secondary antibody controls (Figure S6B). Collectively, our results suggest that GP63 reaches the perinuclear area of the cell shortly after macrophage-parasite contact occurred leading to degradation and cleavage of various members of AP-1 subunits, leading to its inability to dimerize and bind DNA and therefore, altering AP-1 transcriptional activity on genes under its regulation.

### GP63 can directly cleave c-Jun

**Figure 9:**
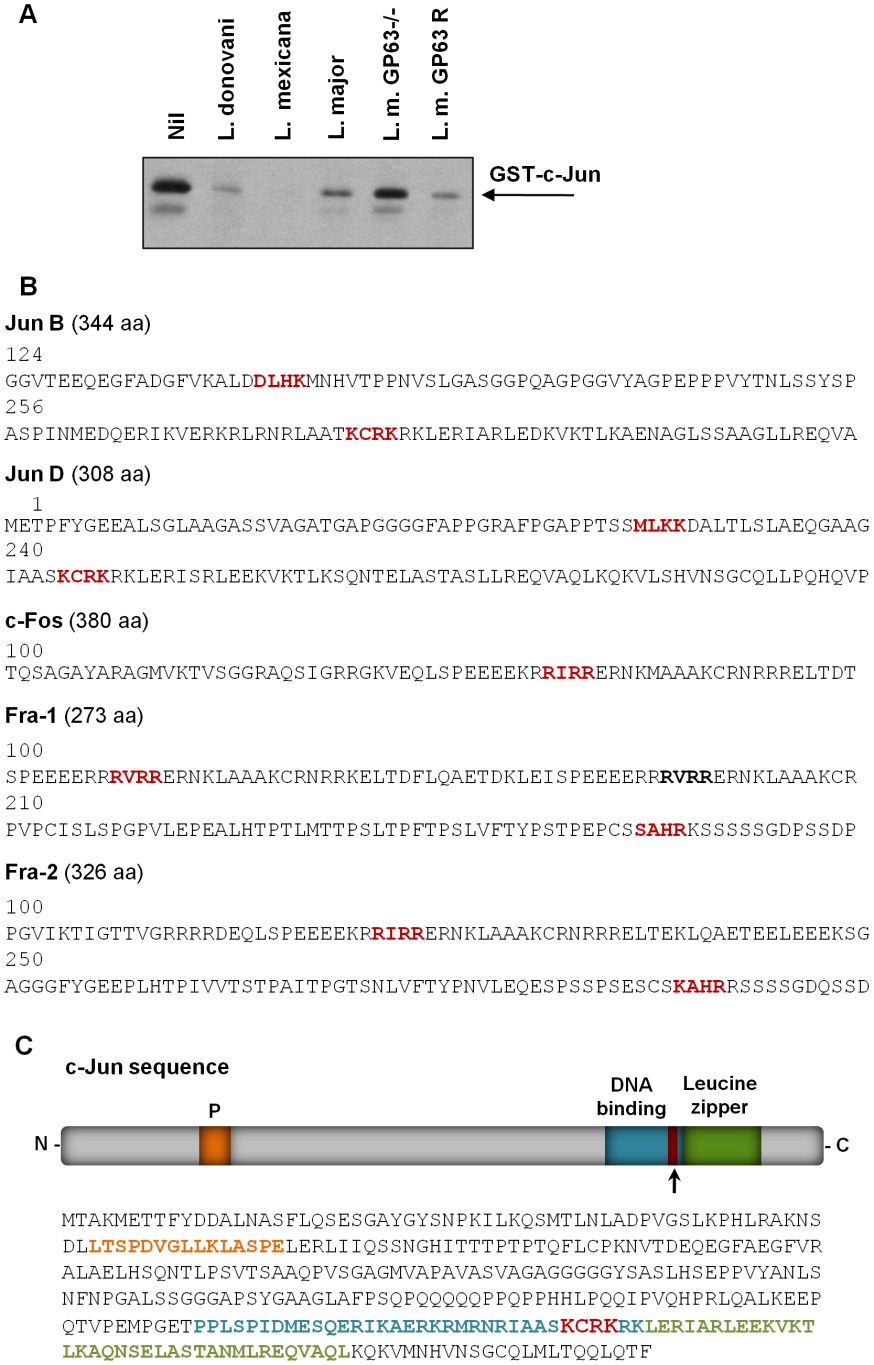
Parasite-free GP63 is sufficient to degrade c-Jun. (**A**) Exogenous GST-c-Jun was incubated with 500 µl of *L. donovani*, *L. mexicana*, *L. major*, *L. major* GP63^−/−^ or *L. major* GP63 Rescued for 30 minutes, and degradation of c-Jun was visualized by WB using anti-GST antibody. (**B**) Jun B, Jun D, c-Fos, Fos B, Fra 1 and Fra 2 protein sequences showing putative GP63 cleavage sites. (**C**) c-Jun sequence analysis showing the putative site of GP63 cleavage.

To further understand the direct effect of GP63 on c-Jun, we used a purified GST tagged-c-Jun protein and incubated it with *Leishmania* promastigotes of different species (including *L. donovani*, *L. mexicana*, *L. major*, *L. major* GP63^−/−^ and *L. major* GP63 Rescued). WB analysis showed that direct contact of parasites expressing GP63 and c-Jun protein is sufficient to induce c-Jun degradation (Figure 9A). This was corroborated by the reduction of c-Jun degradation when incubated with the GP63^−/−^ strain. Figure S7 shows that *L. tarentolae* has no effect on the degradation of GST-c-Jun.

GP63 recognizes a four amino acid motif in its target protein substrates based on amino acid characteristics: polar/hydrophobic/basic/basic amino acids (P_1_- P′_1_-P′_2_-P′_3_) [19]. Sequence analysis of AP-1 subunits revealed putative cleavage sites within c-Jun, Jun B, Jun D and c-Fos (Figure 9B). Of interest, one of the sequence-identified cleavage sites of c-Jun was found between the leucine zipper and the DNA binding domain as shown in Figure 9C. In addition, this motif is found at amino acids 271-275, which will generate cleaved fragments with molecular weight similar to the one detected by WB of lysates from infected cells (∼30 kDa).

Collectively, these data indicate that the *Leishmania* protease GP63 actively participates in altering the DNA binding capacity of AP-1 as a consequence of the diminished expression and cleavage of its subunits. These data further corroborate a mechanism whereby GP63 can enter the cell using lipid raft microdomains, and show for the first time that GP63 reaches the perinuclear area where it proteolytically degrades AP-1 subunits.

## Discussion

*Leishmania* parasites have evolved many mechanisms to undermine macrophage signalling pathways in order to survive and replicate inside these cells. For instance, parasite-mediated activation of macrophage PTPs leads to protein dephosphorylation resulting in the inactivation of transcription factors controlling the expression of many genes required for the effective activation of the innate immune response [36], and macrophage effector functions such as NO production [37]. We have previously reported that *Leishmania* promastigote infection induces degradation and inactivation of some transcription factors. For example, STAT 1 is inactivated by a proteasome mediated mechanism [4], and NF-kB activity is altered in a cleavage-dependent fashion [5]. We show that cleavage of p65 generates an active fragment, p35, which is able to translocate into the nucleus, where it dimerizes with p50 to induce specific chemokine gene expression. Interestingly this cleavage event was found to occur in the macrophage cytoplasm in a GP63-dependent mechanism [5].

Along with STAT and NF-κB, AP-1 is responsible for the transcription of iNOS [38]. NO is a by-product of iNOS-mediated conversion of L-arginine to L-citruline and is essential for the control of *Leishmania* infection [3], [36]. Among other genes regulated by AP-1 in macrophages and known to be affected by *Leishmania* infection are TNFα, IL-1β and IL-12 [24], [25], [26], [39]. The breadth and importance of the immunological functions of AP-1 highlights how detrimental its degradation is to host defence against *Leishmania* infection. Previously, Descoteaux and Matlashewski (1989) demonstrated that the *c-fos* gene, one of the main activators of AP-1, was down-regulated due to abnormal PKC signalling [40]. More recently Ghosh and colleagues (2002) reported *Leishmania*-dependent inactivation of both AP-1 and NF-κB in a ceramide dependent mechanism, where increased levels of intracellular ceramide conducted to the down-regulation of classical PKC activity and impartment of the phosphorylation of ERK, which results in decreased AP-1 activation [10]. These previous reports have given some indication of AP-1 inactivation by *Leishmania*. Here we further demonstrated the molecular mechanisms involved in the AP-1 inactivation by *Leishmania* parasites and its impact on IL-12 expression.

We have found that infection with several *Leishmania* species alters the DNA binding capacity of AP-1. In particular, we have shown that the parasite metalloprotease GP63 is responsible for this DNA binding alteration and is able to induce the degradation/down-regulation and cleavage of c-Jun, the central component of the AP-1 transcription factor [41], as well of other components including c-Fos, Fra-1, Fra-2 and Jun B. We provide evidence that GP63 exerts its effect by its internalization into the host cell, in a mechanism that is independent of parasite internalization, and induces AP-1 proteolysis within the nucleus or in the nuclear membrane.

The present study corroborates along with previous studies (Ghosh *et. al*) that AP-1 is down-regulated by *Leishmania* parasites. Alteration of AP-1 activity varies according to the pathogen, for instance it has been shown, that the hepatitis C virus alters MAP kinases and AP-1 to accelerate the cell cycle progression, helping the development of hepatocellular carcinoma and HCV development [42]. Another example is the Edema toxin produced by *Bacillus anthracis*; this toxin is able to up-regulate macrophage gene expression, among them genes that are known to be involved in inflammatory responses, regulation of apoptosis, adhesion, immune cell activation, and transcription regulation. Interestingly this up-regulation was found to correlate with induced activation of AP-1 and CAAAT/enhancer-binding protein-beta [43]. In contrast with these reports where different pathogens up-regulate AP-1 to survive inside the host cell, herein we have shown how this transcription factor is down-regulated after *Leishmania* infection in a cleavage-dependent manner. Whether AP-1 down-regulation is a general mechanism used by different intracellular protozoa requires further investigation.

The AP-1 transcription factor is formed by dimers of Jun and Fos family members. In addition, the Jun proteins can dimerize with other proteins that share the leucine zipper region such as ATF-1 and ATF-2 [8], [27]. Although we did not test other non-classical AP-1 subunits, we demonstrated that at least 5 of the classical subunits belonging the Jun and Fos families are degraded by the parasite within 1 hr of infection. Of interest, c-Jun subunit, one of the main activators of AP-1 along with c-Fos, is cleaved generating a GP63-mediated 30 kDa fragment. The cleaved product would be unable to dimerize and bind DNA, as it has been demonstrated that truncated c-Jun deprived of either the leucine zipper or the DNA binding domain results in only marginal AP-1 transactivation [41], [44], [45]. The generation of c-Jun fragment by GP63 can explain the lower AP-1 binding activity observed in the EMSA experiment. Furthermore, Fos B, which is not cleaved or degraded and also apparently absent in AP-1 complexes (Figure 2B), lacks putative GP63 cleavage sites. Surprisingly Jun D presents two putative sites of cleavage by GP63. However, we did not detect either complete degradation/down-regulation or cleavage products. One possible explanation is that the structural conformation of this protein renders these sites unavailable for GP63-Jun D interaction.

GP63 is known to interact with various substrates. For instance we have recently reported that *Leishmania* GP63 impacts the stability of cortactin and caspase-3, and negatively regulates p38 kinase activity [23]. Furthermore, we have shown that GP63 cleaves host PTPs resulting in enzymatic activation and leading to JAK 2 dephosphorylation, and inhibition of NO production in IFN-γ primed and infected macrophages [22]. Our current study further supports the important role of GP63 as a negative regulator of host cell functions, actively participating in the pleiotropic effects excreted by *Leishmania* parasite to suppress the immune response, our results showed that internalization of GP63 by cells from innate immune response is independent of parasite internalization, as our data revealed GP63 proteolytic activity was not affected by inhibition of phagocytosis, but clearly abolished by a lipid raft disruptor, strongly suggesting that lipid rafts microdomains are important for internalization of GP63. Proteins that have a GPI anchor have affinity for lipid rafts, and it has been reported that these rafts recognize these GPI anchors allowing the entrance of GPI-bearing proteins in endocytic vesicles [33], [34]. In addition, Brittingham and collaborators showed interaction of GP63 with the fibronectin receptor (α4β1), that also translocate into the lipid rafts microdomains [46], suggesting that GP63 could have two different ways to get access into the cell: 1) GPI-anchor (native and excreted) and 2) receptor mediated (RGP63). Additionally, we have shown that the GPI anchor is important for the internalization of GP63 since recombinant GP63 (rGP63) lacking the GPI anchor is less internalized [22]. Most importantly, GPI anchor seems to be required for the cellular localization of GP63 since rGP63 is localized inside intracellular compartments whereas GPI-GP63 (native protein) is found within nuclear membrane (see Figure 6). Despite this evidence we have not excluded the possibility that GP63 could use other mechanisms to enter the cells, such as micropinocytosis or classical endocytosis pathways.

One of the main finding of this research is the macrophage nuclear localization of GP63. One plausible mechanism is by the recognition of its GPI domain by the recently described lipid microdomains rich in cholesterol and sphingolipids in the nuclear membrane [47]. Another possible mechanism for the internalization of GP63 inside the nucleus is the presence of a nuclear localization signal (NLS)-like motif (Figure S8) in the GP63 sequence. Nuclear proteins are usually transported inside the nucleus by recognition of a NLS, which usually consist of short chains of basic amino acids with the signature motif K-K/R-X-K/R [48]. These NSLs are recognized by the adaptor molecule importin α, which forms a hetorodimer with the transporter receptor importin β. The importin α/β-NSL-cargo complex is then translocated through the nuclear pore complex [48], [49]. The exact mechanisms of how the nuclear translocation of GP63 occurs are currently under investigation.

In summary, GP63 seems to preferentially target AP-1 subunits within the nuclear membrane, altering its DNA binding capacity. Given the critical role of this transcription factor in the transcription of several genes involved in the innate immune response, alterations in AP-1 activity can dramatically contribute to the down-regulation of innate immune functions observed during the early stages of *Leishmania* infection. Therefore, this novel mechanism of evasion by *Leishmania* further demonstrates the complex negative regulatory mechanisms developed by the parasite, which has permitted its adaptation to the harsh intracellular environment leading to its survival and propagation within its mammalian host.

## Materials and Methods

### Cell culture, macrophage infection and reagents

Immortalised murine bone marrow derived macrophages B10R cell line were maintained at 37°C in 5% CO_2_ in Dulbecco's Modified Eagle medium (DMEM) supplemented with 10% heat inactivated FBS (Invitrogen, Burlington, ON, Canada) and 100 U/ml penicillin 100 µg/ml streptomycin and 2 mM of L-glutamine (Wisent, St-Bruno, QC, Canada). *Leishmania* promastigotes (L. *donovani infantum*, *L. donovani* 1S2D, *L. donovani* R2D (LPG ^−/−^), *L. mexicana*, *L. major* A2 (WT), *L. major* GP63 ^−/−^, *L. major* GP63 Rescued [29] and *L. tarentolae*) were grown and maintained at 25°C in SDM-79 culture medium supplemented with 10% FBS by bi-weekly passage. Macrophages were infected at parasite to macrophage ratio 20∶1 with stationary phase promastigotes for the times specified in each Figure legend. Using this ratio of infection we normally observe around 30% and 60% of infected cells in 1 or 2 hr, respectively. When chemical inhibitors were used, 2 µM cytochalasin D (Sigma-Aldrich, St-Louis MO, USA) and 20 mM Methylβ-cyclodextrin (MβCD) (Sigma-Aldrich, St-Louis MO, USA), cells were treated 1 hr prior to infection and the inhibitor remained throughout the infection time.

### Electrophoresis Mobility Shift Assay (EMSA) and supershift assays

B10R macrophages (2×10^6^) were infected, washed three times with Phosphate Buffered Saline (PBS) to remove non-internalized parasites, and processed for nuclear extraction as previously described [4], [50]. Briefly, macrophages were collected in 1 ml of cold PBS, centrifuged and pellets were resuspended in 400 µl of ice-cold buffer A (10 mM HEPES, 10 mM KCl, 0.1 mM EDTA, 0.1 mM EGTA, 1 mM DTT and 0.5 mM of PMSF) and incubated 15 min on ice. Twenty five µl of IGEPAL 10% (Sigma-Aldrich, St-Louis MO, USA) were added, and samples vortexed for 30 sec. Nuclear proteins were pelleted by centrifugation and resuspended in 50 µl of cold buffer C (20 mM HEPES, 400 mM NaCl 1 mM EDTA, 1 mM EGTA 1 mM DTT and 0.5 mM PMSF).

Protein concentrations were determined by Bradford assay (Bio-Rad, Hercules CA, USA). 6 µg of nuclear proteins were incubated for 20 min at room temperature with 1 µl of binding buffer (100 nM Hepes pH 7.9, 8% v/v glycerol, 1% w/v Ficoll, 25 mM KCl, 1 mM DTT, 0.5 mM EDTA, 25 mM NaCl, and 1 µg/µl BSA) and 200 ng/µl of poly (dI-dC), 0.02% bromophenol blue and 1 µl of γ-P^32^labeled oligonucleotide containing a consensus sequence for AP-1 binding complexes (5′-CGTTTGATGACTCAGCCGGAA-3′) (Santa Cruz Biotechnology Inc, Sta Cruz CA, USA). After incubation, DNA-protein complexes were resolved by electrophoresis in non-denaturing polyacrylamide gel 5% (w/v). Subsequently gels were dried and autoradiographed. Competition assays were conducted by adding a 100-fold molar excess of homologous unlabeled AP-1 oligonucleotide, or the non-specific competitor sequence for SP-1 binding (5′-ATTCGAATCGGGGCGGGGCGAGC-3′).

For supershift assay, 2 µg of nuclear protein extract were incubated for 1 hr at room temperature with binding buffer, poly (dI-dC), 0.02% bromophenol blue, labeled oligonucleotide and 4 µg of individual specific antibodies (α-c-Jun, Jun B, Jun D, c-Fos, Fos B, Fra 1 or Fra 2; Santa Cruz Biotech Inc, Sta Cruz CA, USA). Complexes were resolved on standard non-denaturing polyacrilamide gel 5% (w/v).

### Western blot

Infected and non infected cells (1×10^6^) were washed 3 times with PBS and lysed with cold buffer (50 mM Tris (pH 7), 0.1 mM, 0.1 mM EGTA, 0.1% 2-mercaptoethanol, 1% NP-40, 40 µg/ml aproptinin and 20 µg/ml of leupeptin). Proteins were dosed by Bradford (Bio-Rad, Hercules CA, USA), and 30 µg of proteins were separated by SDS-PAGE, and transferred onto PVDF membranes (GE healthcare, Piskataway NJ, USA). Membranes were blocked in 5% non-fat dry milk, washed and incubated for 1 hr with α-c-Jun (BD Biosciences, San Jose, CA, USA), α-Jun B, α-Jun D, α-c-Fos, α-Fos B, α-Fra 1 and α-Fra 2 (Santa Cruz Biotech Inc. Sta Cruz CA, USA). After washing, membranes were incubated 1 hr with α-mouse or α-rabbit HRP-conjugated antibody (GE healthcare, Piskataway NJ, USA), and developed by autoradiography.

### Imunoprecipitation

B10R macrophages (10×10^6^) were infected with either *L. major* A2, or *L. major* GP63 ^−/−^ or *L. major* GP63 Rescued for 1 hr, and nuclear proteins were extracted as previously described in [5]. c-Jun was immunoprecipitated from pre-cleared nuclear extracts with 2 µg antibody, followed by addition of 12.5 µl (packed volume) of protein A/G PLUS agarose beads (Santa Cruz Biotech Inc. Sta Cruz CA USA). Beads were washed three times and bound proteins were analyzed by WB as described above.

### Confocal microscopy

B10R macrophages (0.5×10^6^) were plated ON in glass cover slips. After infection for 30, 60 and 180 min cells were gently washed 3 times with PBS, and then fixed with 4% p-formaldehyde for 30 min at 4°C. Slides were permeabilized for 5 minutes with PBS containing 1% BSA and 0.05% NP-40 and blocked with 5% non-fat dry milk for 1 hr. Incubation with primary antibody α-c Jun or α-c-Fos or α-GP63 mouse monoclonal antibody clone #96 [51] was conducted in humid dark chamber for 1 hr at room temperature. After three washes with PBS, cells were incubated with secondary antibody (Alexa Fluor 488 or 594, from Molecular probes, Burlington ON, Canada) for 1 hr. Nuclei were stained with propidium iodide or DAPI for 10 min and slides were mounted in permaflour medium (Thermo Co. Waltham MA, USA). Images were taken using an Olympus FV1000 confocal microscope and a Zeiss LCS 500.

### IL-12 mRNA expression analysis

B10R macrophages (10×10^6^) were infected with either *L. major* A2, *L. major* GP63^−/−^, *L. major* GP63 Rescued or L. tarentolae for 18 hr or treated with 20 µM of JNK inhibitor SP600125 for 1 hr. Thereafter cells were stimulated with 100 ng/ml of LPS for 6 hr and RNA extracted using TRIzol reagent according to the manufacturer's instruction (Invitrogen Canada). Reverse transcriptase was performed using oligo(dT) primers. Quantitative relative PCR was performed using Invitrogen Platinum qPCR Super-Mixes and 0.4 µM primer in 25 µl and the following parameters: 50°C for 2 min and 95°C for 3 min (95°C for 20 s, 60°C for 30 s, 72°C for 20 s, 80°C (reading step) for 20 s) for 40 cycles followed by a melting curve. Annealing temperature was 60°C. IL-12 primer sequences: 5′-GGA AGCACG GCA GCA GAA TA-3′ and 3′-AAC TTG AGG GAG AAG TAGGAA TGG-5′.

## Supporting Information

Figure S1 LPS-induced IL-12 expression is regulated by *Leishmania* in a GP63 dependent manner. B10R macrophages were infected (1∶20 ratio) with *L. major*, *L. major* GP63^−/−^, *L. major* GP63 Rescued or *L. tarentolae* stationary promastigotes for 18 hr or treated with 20 µM of JNK/c-Jun inhibitor - SP600125. After infection, mRNA was extracted and submitted to qRT-PCR. Data shows mean ± SEM of three different experiments.
(0.25 MB TIF)

Figure S2 Efficacy of cytochalasin D and cytotoxicity of MβCD. (A) % of infected cells pre-treated with 2 µM/ml of cytochalasin D 1 hr before infection with *L. mexicana* (1 hr infection) (B) Cytotoxicity of MβCD in B10R macrophages treated for 1 hr with indicated doses of MβCD using the XTT metabolizing assay.
(0.24 MB TIF)

Figure S3 GP63 partially co-localizes with lipid raft domains. (A) B10R macrophages were incubated with *L. major* supernatant for 1 hr and then stained for confocal microscopy to evaluate co-localization of lipid rafts domains and GP63. The lipid raft marker cholera toxin B (CTxB) is shown in red and GP63 is labelled in green (Alexa 488). (B) B10R macrophages were infected with *L. major* for 1 hr and then stained with unspecific anti-mouse IgG2a followed by anti-mouse Alexa 488 or infected and then incubated with anti-mouse Alexa 488.
(0.68 MB TIF)

Figure S4 Controls for c-Jun cleavage and GP63 activity. (A) B10R macrophages were incubated for 5, 30, 60 and 120 minutes with MβCD (20 µM) and total cell lysates were subjected to WB against c-Jun. (B) B10R macrophages were infected with stationary phase promastigotes for 5, 30 and 60 minutes, after infection cells were washed, lysed with 100 µl of SLB 1× and boiled for 5 minutes. Samples were subjected to WB against c-Jun. (C) B10R macrophages were infected as in (B) and cells were lysed with lysis buffer containing 1 mM of phenantroline. Total cell extracts were subjected to WB against c-Jun. (D) *L. mexicana* supernatant was pre-treated with 1 mM of phenanthroline (phen) for 1 hr. After, supernatant from *L. mexicana* (mex), *L. mexicana* + phen, *L. tarentolae* (Tar), *L. major* (Mj), *L. major* GP63^−/−^ (KO) were added to B10R macrophages and incubated for 1 hr. Total cell extracts were subjected to WB against c-Jun, GP63 and actin. For all the figures actin was used as loading control.
(0.66 MB TIF)

Figure S5 Purity of cytoplasmic and nuclear fractions. B10R macrophages were infected with *L. major*, *L. major* GP63^−/−^ or *L. major* GP63 Rescued stationary promastigotes for 1 hr. After infection, cytoplasmic and nuclear proteins were separated and subjected to WB against lysosomal marker LAMP-1, ER specific protein KDEL receptor, actin, histone and GP63 antibodies.
(0.39 MB TIF)

Figure S6 Negative control to confocal experiments. B10R macrophages were infected with *L. major* and cells were stained with unspecific anti-mouse (IgG2a) or anti-rabbit antibodies followed by secondary antibody (Alexa 488 or Alexa 546 respectively) or only stained with anti-mouse (Alexa 488) or anti-rabbit (Alexa 546) antibodies.
(1.27 MB TIF)

Figure S7 *L. tarentolae* has no effect over recombinat c-Jun. Exogenous GST-c-Jun was incubated with 500 µl of *L. donovani*, *L. mexicana*, or *L. tarentolae* for 30 min and degradation of c-Jun was visualized by WB using anti-GST antibody.
(0.32 MB TIF)

Figure S8 NLS-like motif in GP63 sequence. GP63 amino acid sequence of *L. mexicana* with the putative NLS (nuclear localization signals)-like motif.
(0.27 MB TIF)
