# Supplementary figures and images for: Leishmania-Induced Inactivation of the Macrophage Transcription Factor AP-1 Is Mediated by the Parasite Metalloprotease GP63
Source: PLoS Pathog. 2010 Oct 14;6(10):e1001148. doi: 10.1371/journal.ppat.1001148 (PMC2954837; doi:10.1371/journal.ppat.1001148)

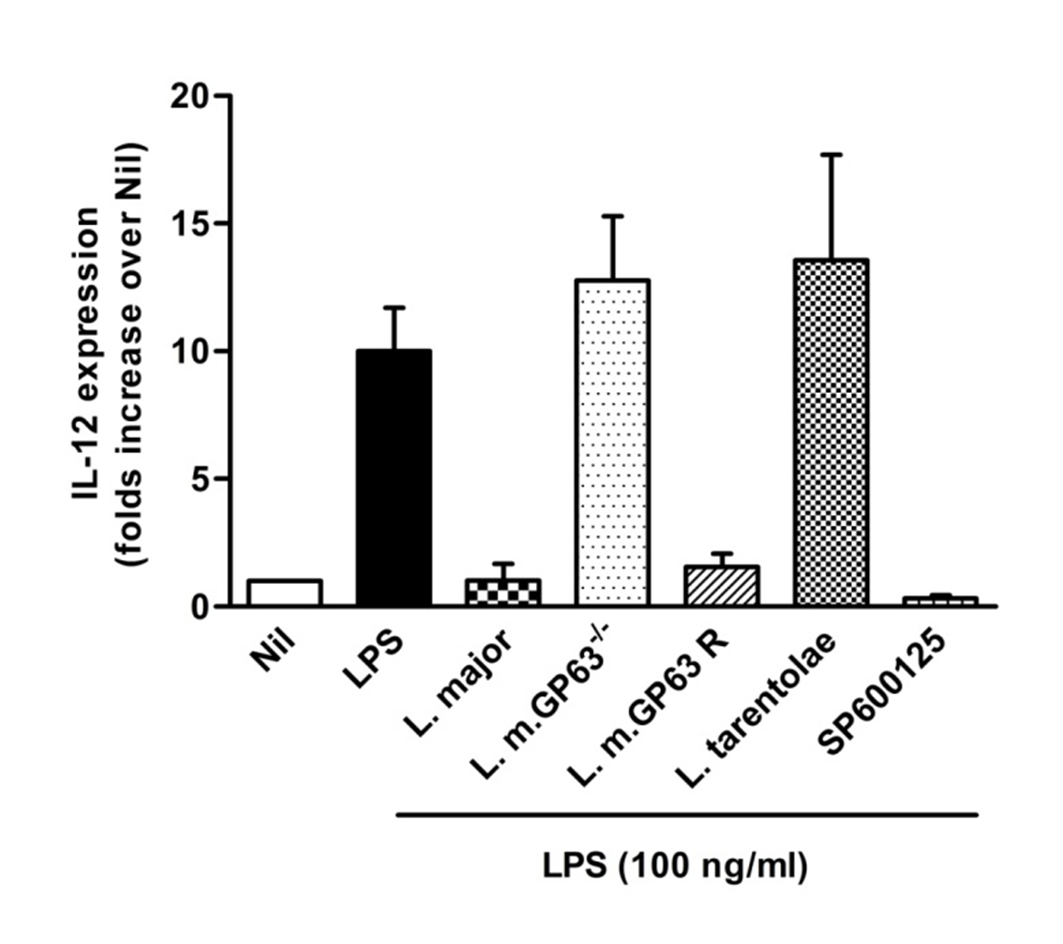

Supplement: Figure S1 — LPS-induced IL-12 expression is regulated by Leishmania in a GP63 dependent manner. B10R macrophages were infected (1∶20 ratio) with L. major, L. major GP63−/−, L. major GP63 Rescued or L. tarentolae stationary promastigotes for 18 hr or treated with 20 µM of JNK/c-Jun inhibitor - SP600125. After infection, mRNA was extracted and submitted to qRT-PCR. Data shows mean ± SEM of three different experiments. (0.25 MB TIF) [file ppat.1001148.s001.tif]

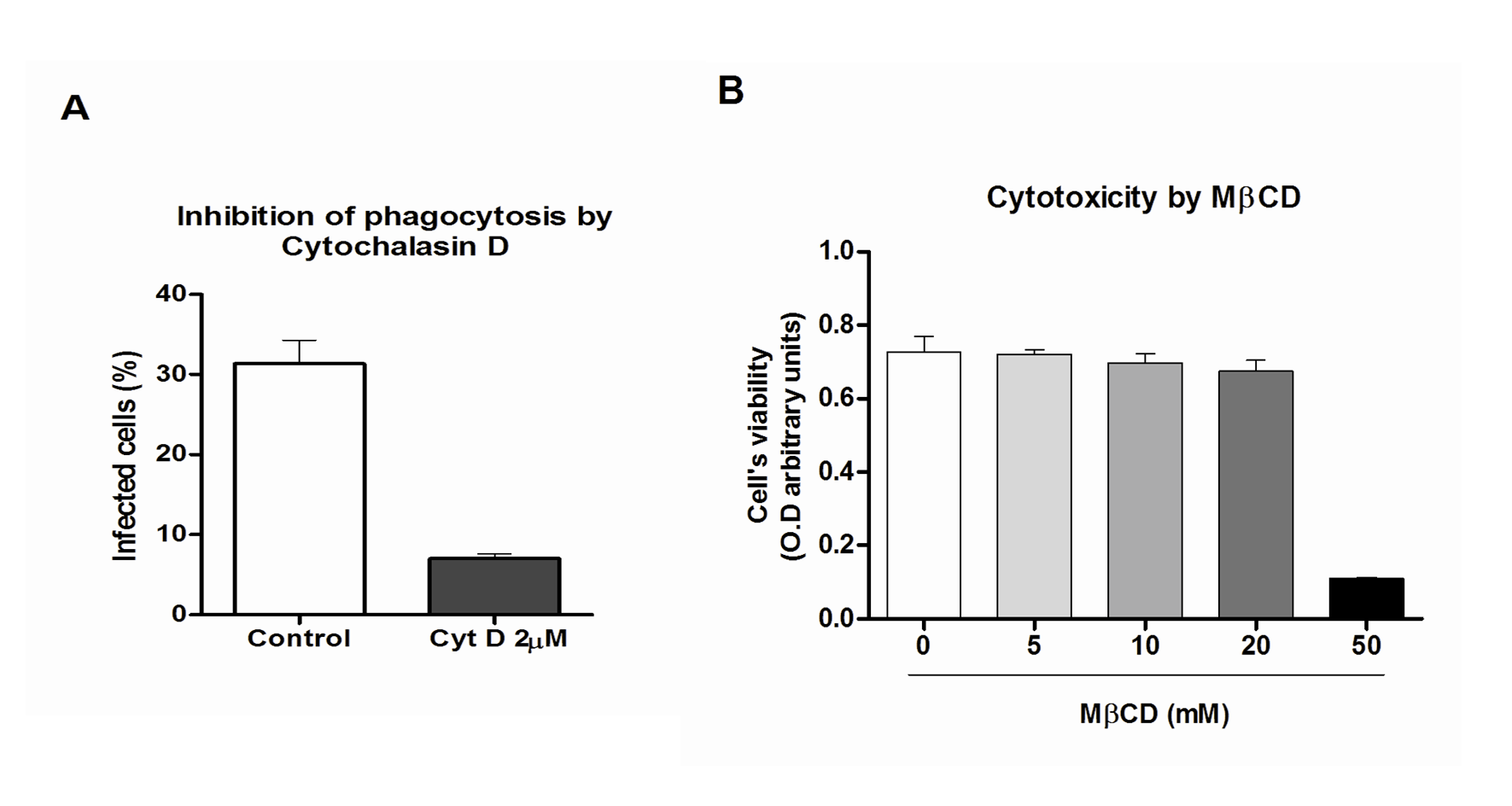

Supplement: Figure S2 — Efficacy of cytochalasin D and cytotoxicity of MβCD. (A) % of infected cells pre-treated with 2 µM/ml of cytochalasin D 1 hr before infection with L. mexicana (1 hr infection) (B) Cytotoxicity of MβCD in B10R macrophages treated for 1 hr with indicated doses of MβCD using the XTT metabolizing assay. (0.24 MB TIF) [file ppat.1001148.s002.tif]

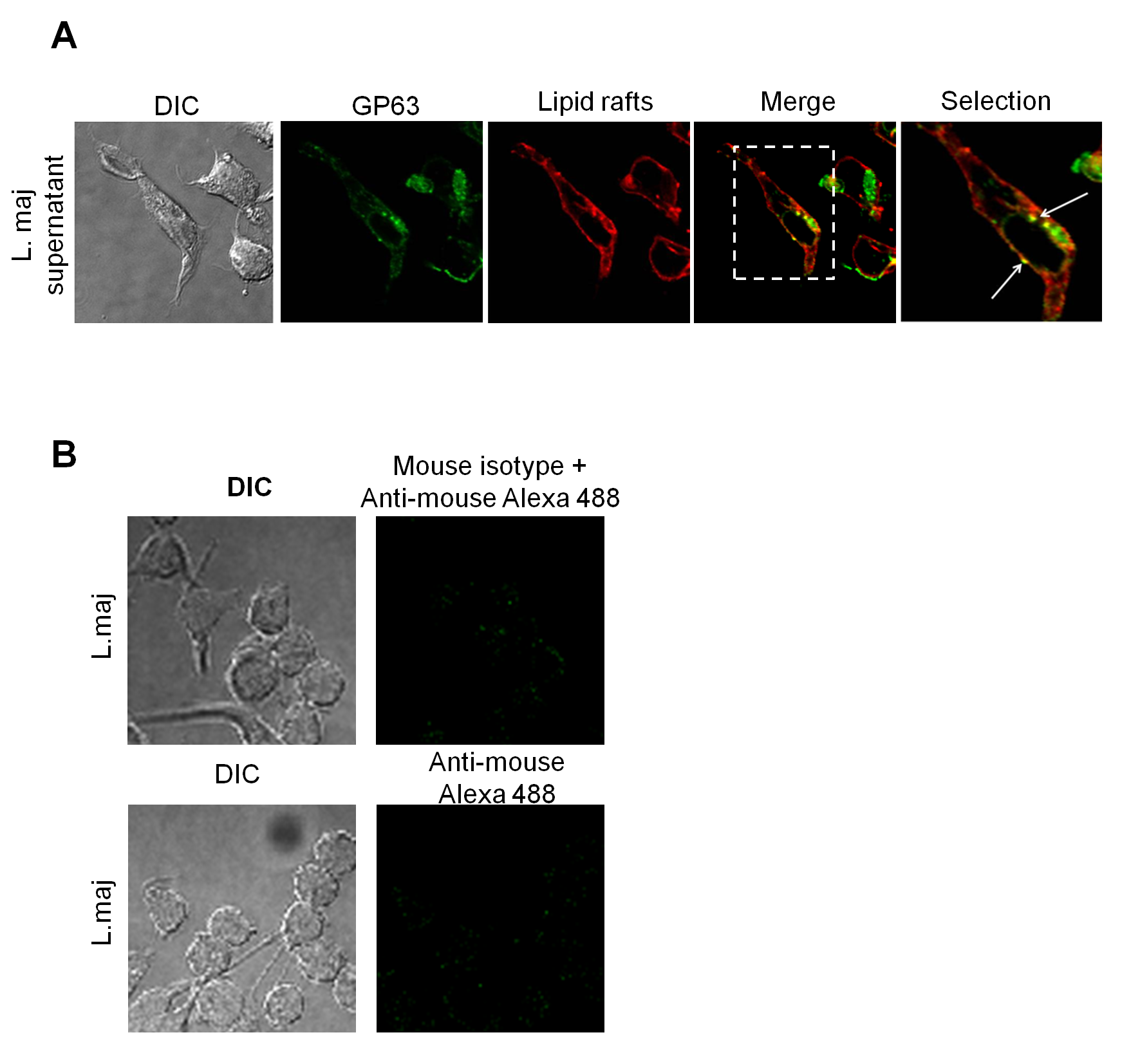

Supplement: Figure S3 — GP63 partially co-localizes with lipid raft domains. (A) B10R macrophages were incubated with L. major supernatant for 1 hr and then stained for confocal microscopy to evaluate co-localization of lipid rafts domains and GP63. The lipid raft marker cholera toxin B (CTxB) is shown in red and GP63 is labelled in green (Alexa 488). (B) B10R macrophages were infected with L. major for 1 hr and then stained with unspecific anti-mouse IgG2a followed by anti-mouse Alexa 488 or infected and then incubated with anti-mouse Alexa 488. (0.68 MB TIF) [file ppat.1001148.s003.tif]

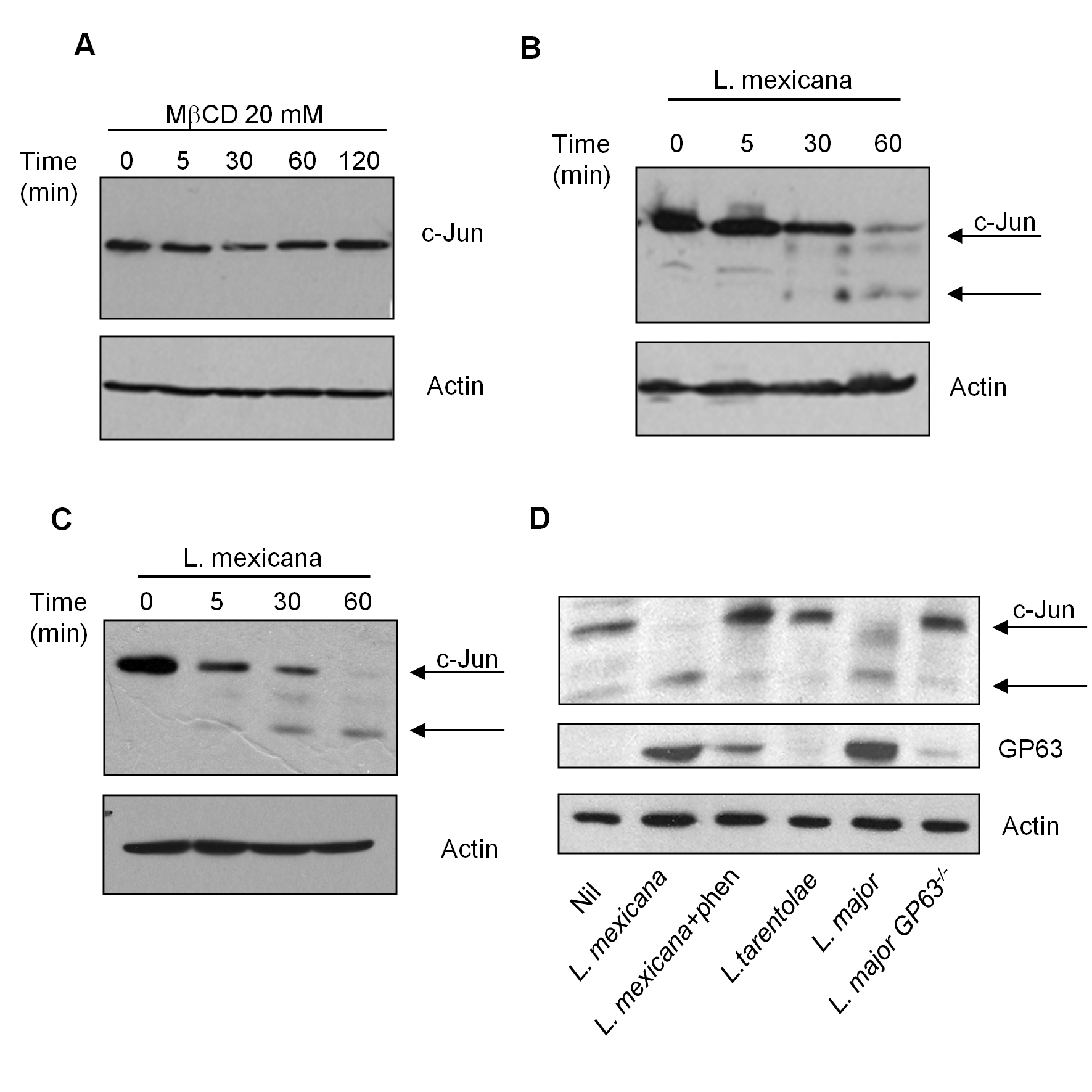

Supplement: Figure S4 — Controls for c-Jun cleavage and GP63 activity. (A) B10R macrophages were incubated for 5, 30, 60 and 120 minutes with MβCD (20 µM) and total cell lysates were subjected to WB against c-Jun. (B) B10R macrophages were infected with stationary phase promastigotes for 5, 30 and 60 minutes, after infection cells were washed, lysed with 100 µl of SLB 1× and boiled for 5 minutes. Samples were subjected to WB against c-Jun. (C) B10R macrophages were infected as in (B) and cells were lysed with lysis buffer containing 1 mM of phenantroline. Total cell extracts were subjected to WB against c-Jun. (D) L. mexicana supernatant was pre-treated with 1 mM of phenanthroline (phen) for 1 hr. After, supernatant from L. mexicana (mex), L. mexicana + phen, L. tarentolae (Tar), L. major (Mj), L. major GP63−/− (KO) were added to B10R macrophages and incubated for 1 hr. Total cell extracts were subjected to WB against c-Jun, GP63 and actin. For all the figures actin was used as loading control. (0.66 MB TIF) [file ppat.1001148.s004.tif]

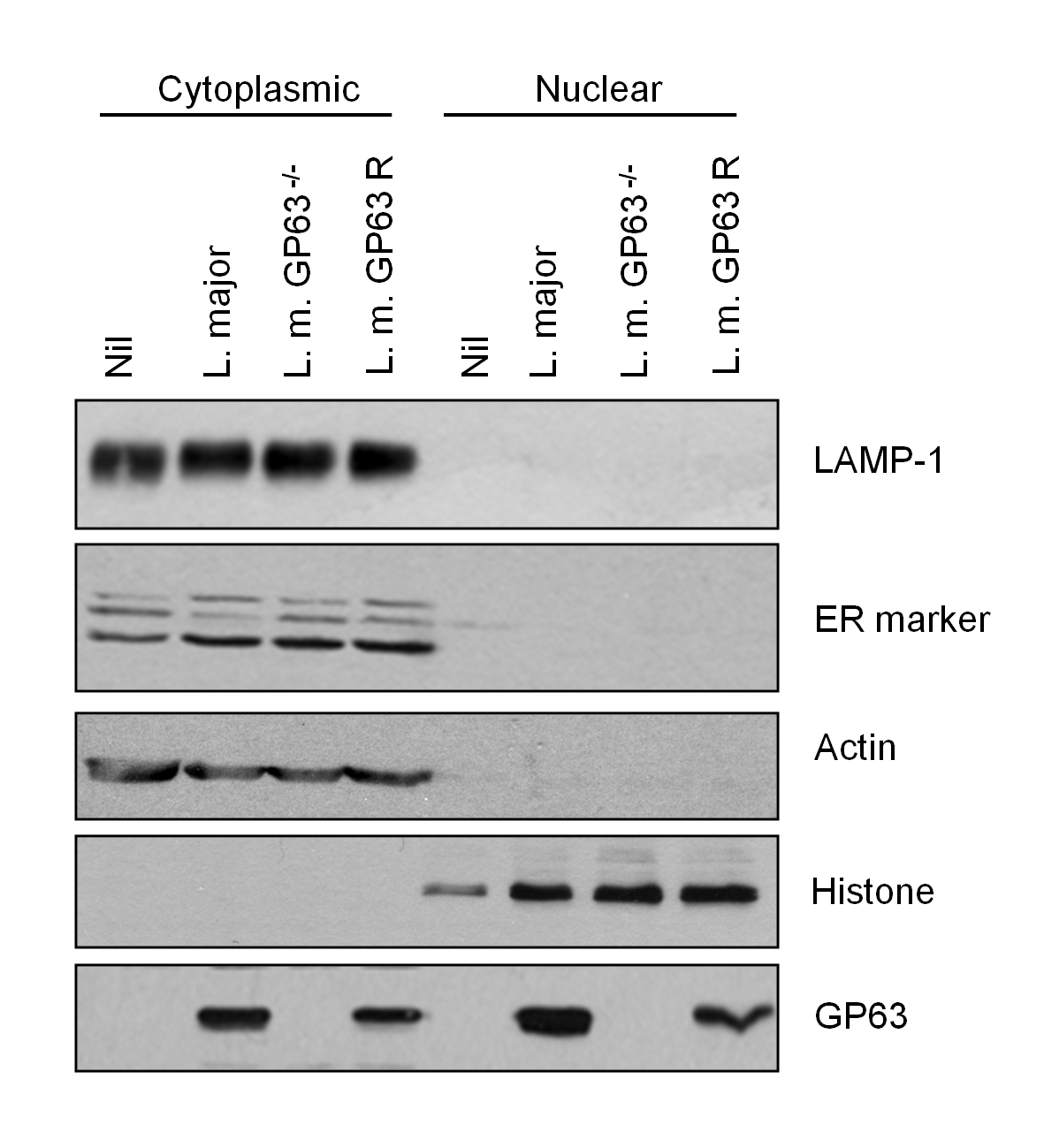

Supplement: Figure S5 — Purity of cytoplasmic and nuclear fractions. B10R macrophages were infected with L. major, L. major GP63−/− or L. major GP63 Rescued stationary promastigotes for 1 hr. After infection, cytoplasmic and nuclear proteins were separated and subjected to WB against lysosomal marker LAMP-1, ER specific protein KDEL receptor, actin, histone and GP63 antibodies. (0.39 MB TIF) [file ppat.1001148.s005.tif]

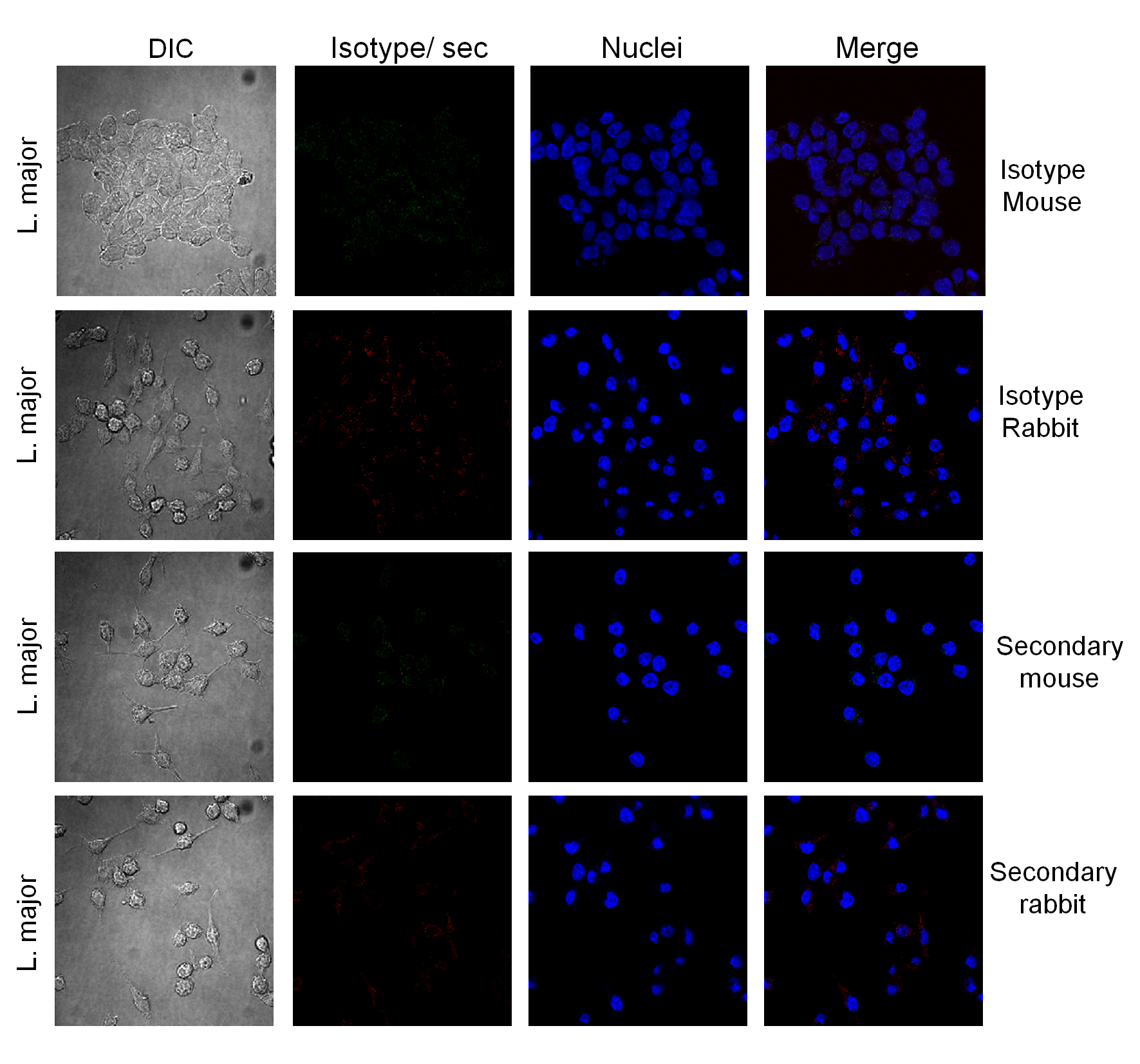

Supplement: Figure S6 — Negative control to confocal experiments. B10R macrophages were infected with L. major and cells were stained with unspecific anti-mouse (IgG2a) or anti-rabbit antibodies followed by secondary antibody (Alexa 488 or Alexa 546 respectively) or only stained with anti-mouse (Alexa 488) or anti-rabbit (Alexa 546) antibodies. (1.27 MB TIF) [file ppat.1001148.s006.tif]

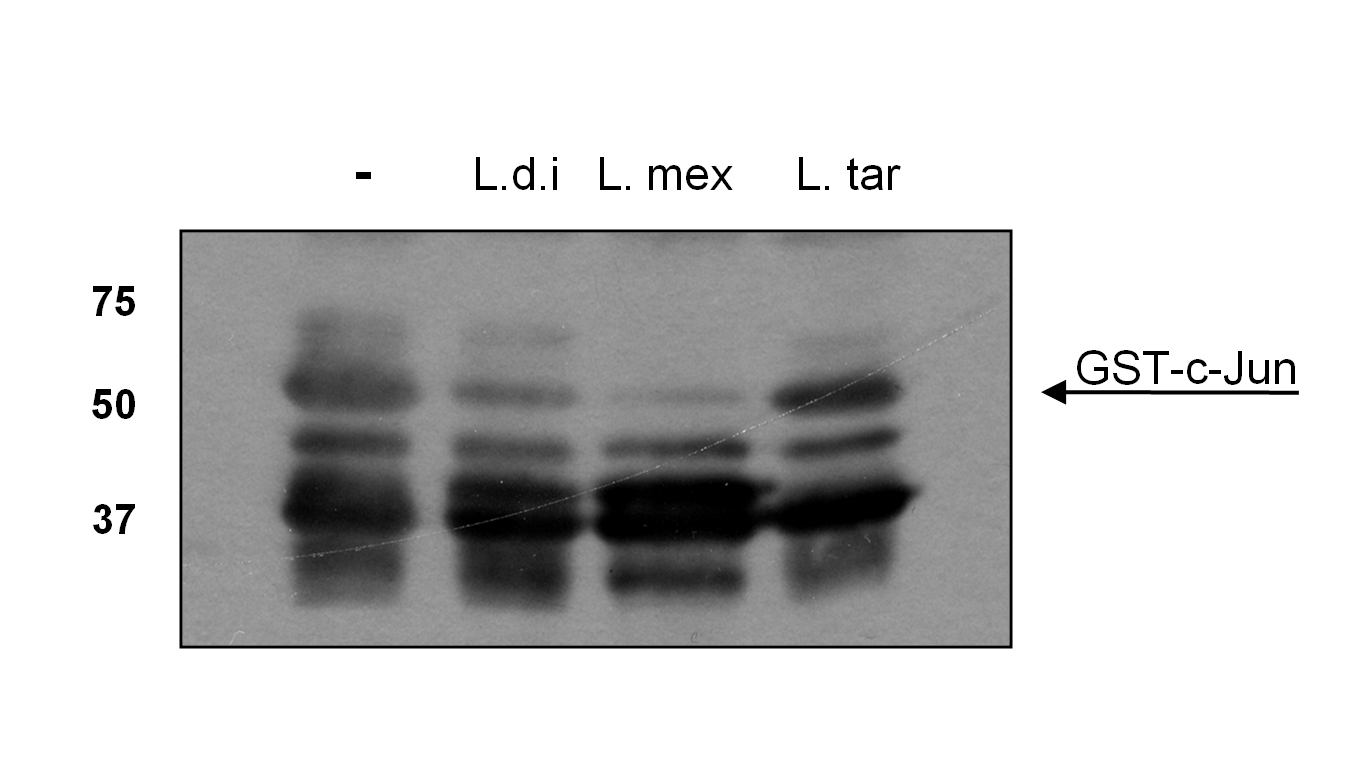

Supplement: Figure S7 — L. tarentolae has no effect over recombinat c-Jun. Exogenous GST-c-Jun was incubated with 500 µl of L. donovani, L. mexicana, or L. tarentolae for 30 min and degradation of c-Jun was visualized by WB using anti-GST antibody. (0.32 MB TIF) [file ppat.1001148.s007.tif]

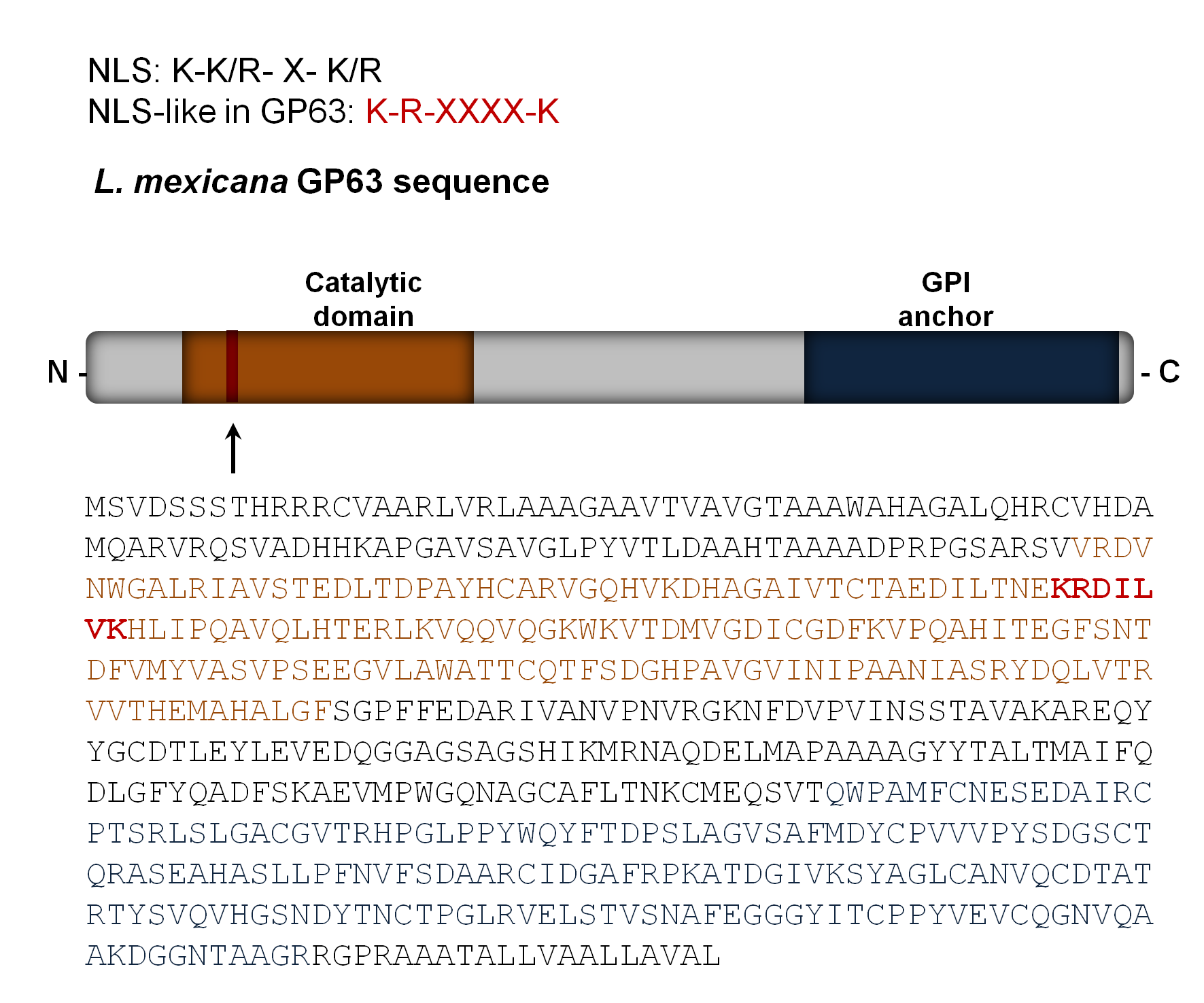

Supplement: Figure S8 — NLS-like motif in GP63 sequence. GP63 amino acid sequence of L. mexicana with the putative NLS (nuclear localization signals)-like motif. (0.27 MB TIF) [file ppat.1001148.s008.tif]
